# The Influence of Ionic Liquids Adsorption on the Electronic and Optical Properties of Phosphorene and Arsenene with Different Phases: A Computational Study

**DOI:** 10.3390/molecules27082518

**Published:** 2022-04-13

**Authors:** Lin Zhu, Aiping Fu

**Affiliations:** 1 College of Chemistry and Chemical Engineering, Qingdao University, Qingdao 266071, China; linzhu960121@163.com; 2 State Key Laboratory of Bio-Fibers and Eco-Textiles, Qingdao University, Qingdao 266071, China

**Keywords:** DFT study, ionic liquids, phosphorene, arsenene, α- and β-phases

## Abstract

Density functional theory (DFT) calculations have been performed to investigate the interfacial interactions of ionic liquids (ILs) on the α- and β-phases of phosphorene (P) and arsenene (As). Nine representative ILs based on the combinations of 1-ethyl-3-methylimidazolium ([EMIM]^+^), N-methylpyridinium ([MPI]^+^), and trimethylamine ([TMA]^+^) cations paired to tetrafluoroborate ([BF_4_]^−^), trifluoromethanesulfonate ([TFO]^−^), and chloridion (Cl^−^) anions were used as adsorbates on the 2D P and As nanosheets with different phases to explore the effect of IL adsorption on the electronic and optical properties of 2D materials. The calculated structure, adsorption energy, and charge transfer suggest that the interaction between ILs and P and As nanosheets is dominated by noncovalent forces, and the most stable adsorption structures are characterized by the simultaneous interaction of the cation and anion with the surface, irrespective of the types of ILs and surfaces. Furthermore, the IL adsorption leads to the larger change in the electronic properties of β-phase P and As than those of their α-phase counterparts, which demonstrates that the adsorption properties are not only related to the chemical elements, but also closely related to the phase structures. The present results provide insight into the further applications of ILs and phosphorene (arsenene) hybrid materials.

## 1. Introduction

The unique and superior physical and chemical properties of two-dimensional (2D) materials make them very important for the development of a new generation of smart electronics, optoelectronics, and new energy devices [1,2,3,4,5,6]. Over the past decades, the research on various 2D layered nanomaterials has grown rapidly, including elemental olefins, transition metal sulfides, transition metal carbides, oxides, and other materials [1]. Recently, due to the discovery of black phosphorene and its excellent electrochemical properties, two-dimensional materials (including P, AS, Sb, Bi) of the 15th group have received great attention [3,7]. Intensive theoretical and experimental efforts have been devoted to exploring their promising properties and potential applications in various fields, such as field effect devices and energy storage devices [8,9,10,11,12,13,14,15,16].

Ionic liquids (ILs) have been widely investigated as novel solvents, electrolytes, and soft functional materials because of their excellent properties, including high ionic conductivity, wide electrochemical windows, and good thermal stability [17,18,19,20,21,22,23,24,25,26,27]. Moreover, the chemical and physical properties of ILs can be modulated by choosing different combinations of cations and anions, so ionic liquids are often called “designer solvents” because of their versatile properties. One of the practical applications of ILs is their utilization as the electrolytes in electrochemical energy-storage devices, such as batteries, supercapacitors, fuel cells, and other related electrochemical reactions, in which 2D materials always act as electrodes [18,28,29,30,31]. Furthermore, ionic liquids can also be used as solvents for liquid-assisted exfoliation pathways for the production of 2D materials [31,32,33,34,35,36,37]. Single- or few-layer 2D nanosheets of graphene and black phosphorous have been successfully exfoliated with specific ILs [32,37]. Besides the applications in the fields of electrochemistry and exfoliation, the ILs/2D composites also show great potential in surface catalysis and gas separation [1,5,7]. Hence, all of the above-mentioned representative applications which combine the novel properties of 2D materials and ILs concern the interfacial structure between surfaces and ILs. Therefore, a better understanding of the ILs interaction with 2D surfaces is crucial for promoting the performance of the related energy storage devices, the efficiency of the exfoliation process, and the activity of the surface catalysis.

Several groups have performed systematic DFT studies on the adsorption behaviors of different types of ILs on common 2D materials, including graphene (carbon nanotubes), h-BN, silicene, germanene, etc. [28,29,30,31,32,33,34,35,36,37,38,39,40,41,42,43,44,45]. Notably, most of the theoretical research focuses on the interfacial behavior of ionic liquids with graphene and its derivatives [28,38,39,40,41,42]. It should be noted that a few theoretical efforts have been made to probe the properties of hybrids composed of ILs and the recently emerged 15th group of elemental 2D nanosheets of P, As, Sb, and Bi. For instance, Lust’s group has reported their preliminary computations of 1-butyl-4-methylpyridinium tetrafluoroborate (BMPyBF_4_) adsorption on Bi (111) surface to collaborate with their experimental observations [46,47]. Lu et al. reported their first-principles investigation of the interfacial interactions and structures of imidazolium-based ILs on a black phosphorus surface [48]. Very recently, we studied the adsorption behavior of [BF_4_]^−^ anion-based ionic liquids on phosphorene (α), arsenene (β), and antimonene (β) [49]. Although the above-mentioned studies have provided some descriptions concerning the interface of ILs on 2D nanosheets of P, As, Sb, and Bi, the deficiency in those studies is the that their selections of ILs are limited. Furthermore, for the 15th group of monolayers, different allotropes, e.g., five typical honeycomb (α, β, γ, δ, and ε) and four non-honeycomb (ζ, η, θ, and ι) structures, have been predicted by Zhang et al. [11]. Altering the phase would make significant changes in their chemical and physical properties. Since the existing literature demonstrates that the interfacial structure and interaction strength correlate with the nature of the surfaces and the ILs, a systematic, comparative study of the interaction between ILs of various shapes and sizes and the 15th group of 2D nanosheets with different phase structures is essential for the selection and evaluation of ILs and 2D materials to improve their performance in various applications. Therefore, in the present paper, the structural and optoelectronic properties of nine representative ILs adsorbed on phosphorene and arsenene were systematically studied via the application of density functional theory (DFT) calculations. The impact of phase selection (of two common allotropes of the α- and β-phases) of P and As on the properties of adsorption systems were explored. The nine representative ILs are based on combinations of three popularly used cations, including 1-ethyl-3-methylimidazolium ([EMIM]^+^), N-methylpyridinium ([MPI]^+^), and trimethylamine ([TMA]^+^), paired to three anions of different shapes and sizes e.g., tetrafluoroborate ion ([BF_4_]^−^), trifluoromethanesulfonate ion ([TFO]^−^), and chloridion (Cl^−^). The above-mentioned ILs were selected because they have been widely used in various applications, such as energy storage devices, sensors, and catalysis [23,24,25]. Moreover, the ILs based on those cations and anions emerge as subjects in many theoretical studies on the interaction of ILs with various surfaces, which can provide reference for comparisons [19,20,21,22,23,24,25,26,27,28,29,30,31,32,33,34,35,36,37,38,39,40,41,42,43,44,45,46,47,48,49]. The effects of cation and anion types on the interfacial interaction parameters involving structures, adsorption energies, densities of states, charge transfers, and optical properties were analyzed. We hope that our calculated results can shed light on the effective screening and design of ionic liquids/2D materials to improve electrochemical device performance, material dispersion, and surface catalytic activity.

## 2. Results and Discussion

### 2.1. Structures and Energies of IL/2D Sheets

**Scheme 1:**
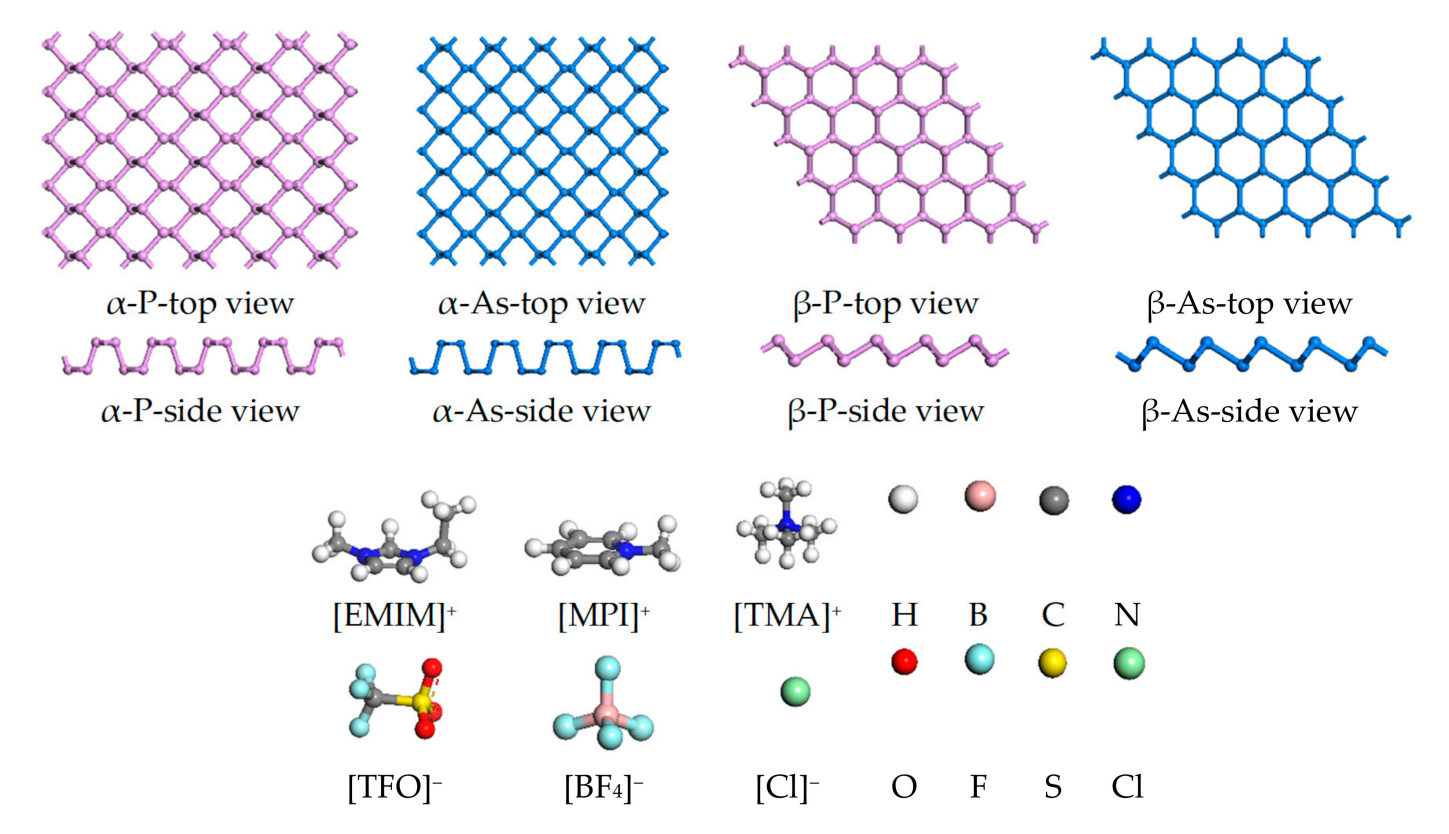
Top and side views of the most stable models of α-P, α-As, β-P, and β-As nanosheets. The structures of cations and anions of ILs.

**Figure 1:**
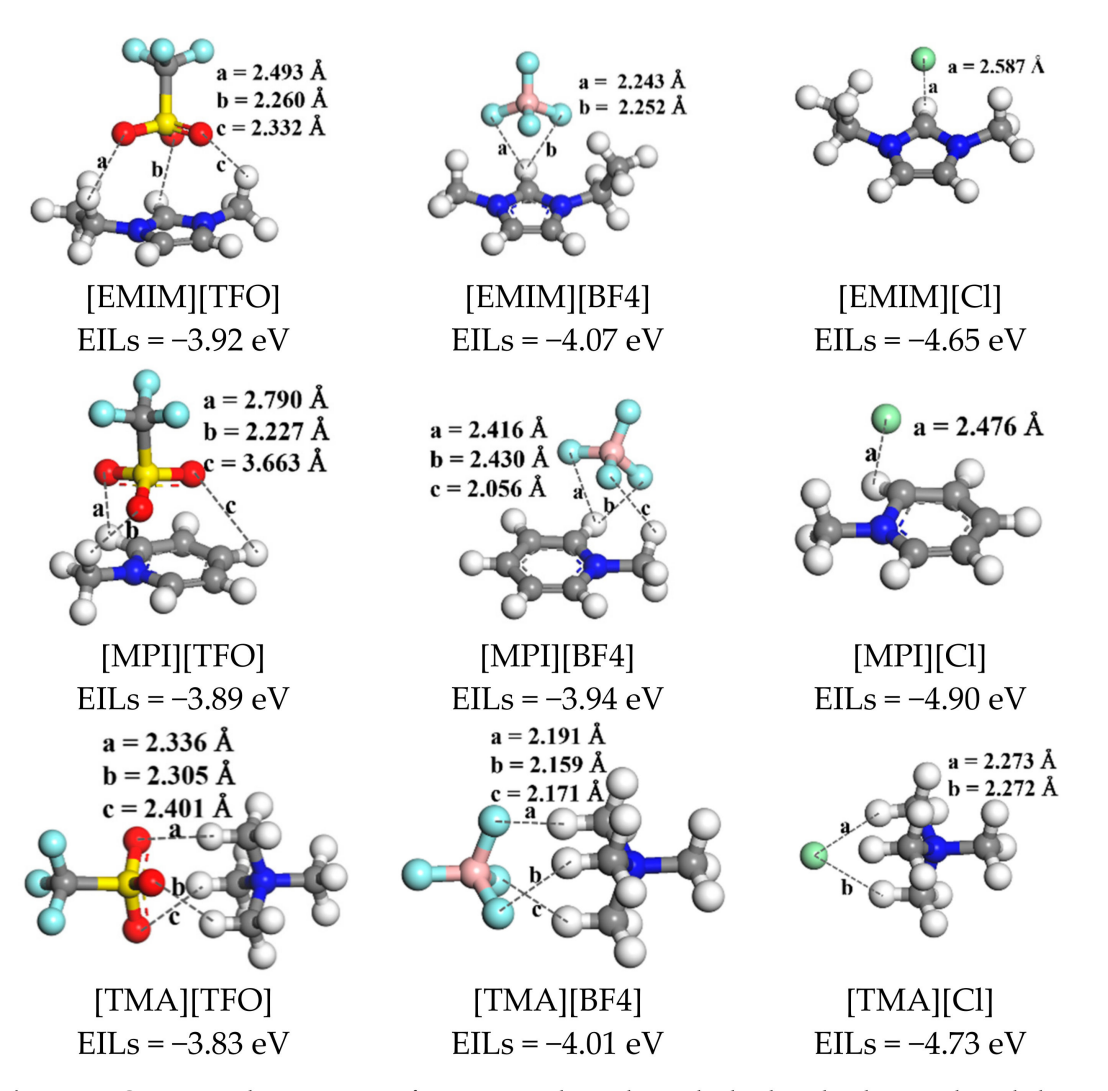
Optimized geometry of nine ionic liquids with the key hydrogen-bond distances (Å) and interaction energy between cations and anions (E_ILs_).

It is well-known that typical allotropes of the 15th group of 2D nanomaterials exhibit structural diversity [2,4]. The optimized structures of phosphorene and arsenene with α- and β-phases are shown in Scheme 1. These four nanosheets all have the graphene-like honeycomb structure. Unlike planar graphene, α-P and α-As (black phosphorene and black arsenene) hold the unique, puckered layered structure, while the β-P and β-As (blue phosphorene and grey arsenene) prefer the buckled layered structure as reported by Zhang et al. [2]. Besides, α-P and α-As have strong in-plane anisotropy. The structures of three cations and three anions of considered ILs with different sizes and shapes are also illustrated in Scheme 1. The geometries of nine ILs based on the combination of the above-mentioned cations and anions ([EMIM][TFO], [EMIM][BF_4_], [EMIM][Cl], [MPI][TFO], [MPI][BF_4_], [MPI][Cl], [TMA][TFO], [TMA][BF_4_], and [TMA][Cl]) were fully optimized, and their binding energies were calculated. Figure 1 shows the most stable geometric structures of ionic liquids, and the interaction distances between the hydrogen atoms of [EMIM]^+^, [MPI]^+^, [TMA]^+^ and the O, F, and Cl atoms of the anion are also marked. For the ion pairs based on [EMIM]^+^, [MPI]^+^ cations, and [TFO]^−^, [BF_4_]^−^ anions, the H on the aromatic ring, the methyl and ethyl groups can be combined with the O atom in [TFO]^−^ and the F atom in [BF_4_]^−^ through hydrogen bonding. For example, the distances of H^…^O in [EMIM][TFO] are 2.493, 2.260, and 2.332 Å, respectively, and the hydrogen bond between the cation and anion is mainly formed by the acidic hydrogen on the carbon atom between the two nitrogen atoms on the imidazole ring. In [EMIM][Cl] and [MPI][Cl], [Cl]^−^ also mainly interacts with the acidic C-H near the N atom on the aromatic ring. For the [TMA]^+^-based ionic liquid, the anion interacts with the methyl group in the cation. For instance, in [TMA][TFO] ionic liquid, the distance between the H on the methyl group of the cation and the O on the anion is 2.336, 2.305, 2.401 Å. Although the interaction between the cation and the anion is mainly governed by the Coulomb force, the optimized structure shows that the hydrogen bond also plays an important role. By comparing the binding energies between the anion and cation, the strength order is [X][Cl] > [X][BF_4_] > [X][TFO]. The strongest interaction is found in [X][Cl], which can be attributed to the small size of anion, resulting in the more compact structures of ILs.

**Figure 2:**
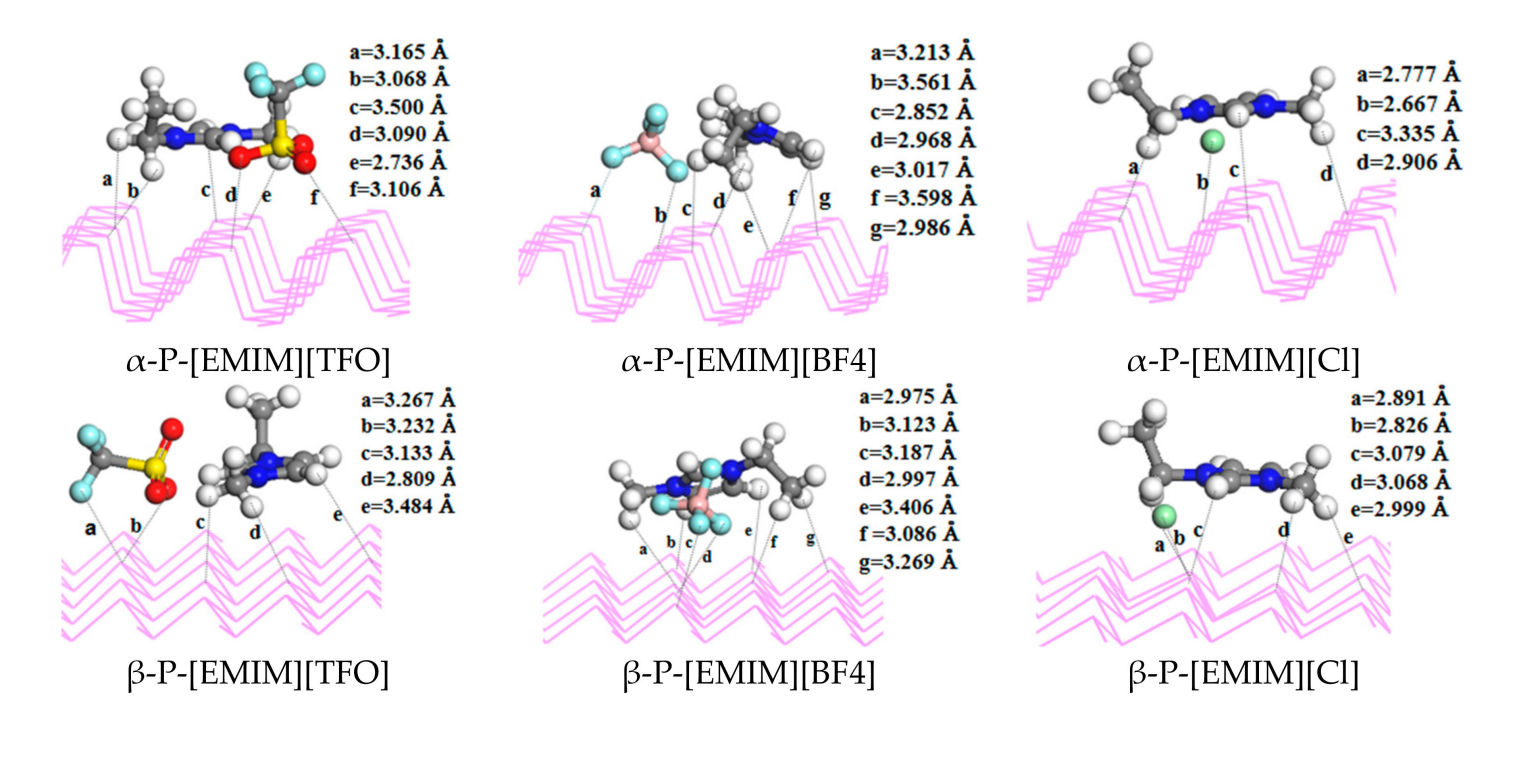

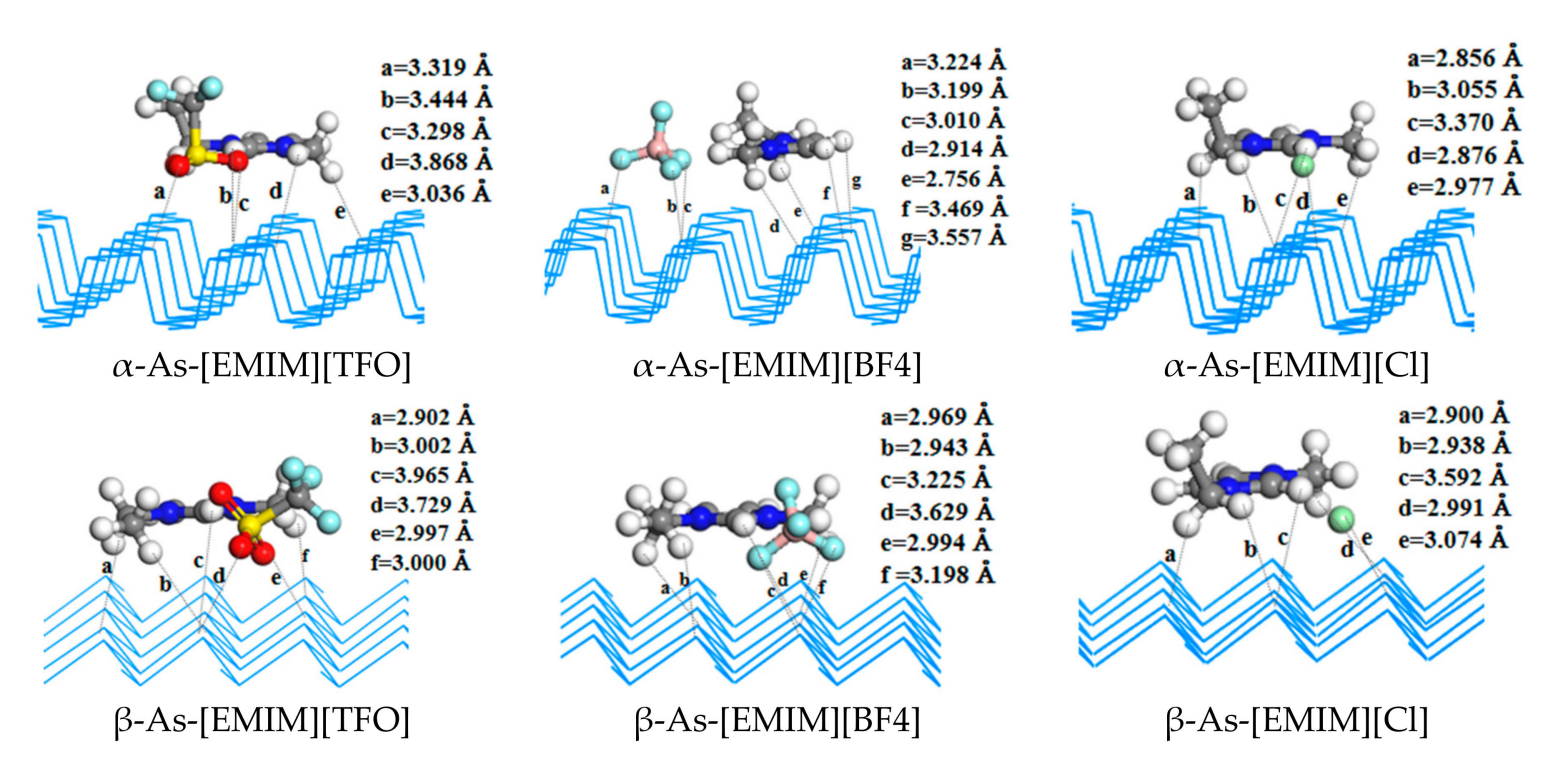
Most stable geometry of ionic liquids adsorbed on 2D surfaces with key interaction distances (Å).

In order to study the interaction mechanism of ionic liquids with α- and β-phase phosphorene and arsenene, in line with previous reports [28,29,30,31,32,33,34,35,36,38,39,40,41,42,43,44,45,46,47,48,49], we considered various orientations and configurations of ionic liquids adsorbed on surfaces, and the initial conceiving structures represented by [EMIM][BF_4_] and [TMA][BF_4_] adsorbed on α-P are shown in Figure S1. The most stable geometries of the ILs 2D adsorption system with critical interaction distances are presented in Figure 2 and Figure S2, and the corresponding adsorption energies are shown in Table 1.

As shown in Figure 2 and Figure S2, both cations and anions prefer to make contact with the nanosheets simultaneously. The moiety of imidazolium- and pyridinium-based cations lie nearly parallel to the sheet because of the π stacking interaction between the aromatic rings and the surface. The non-aromatic [TMA]^+^ cation interacts with the surface through the methyl group. The contact between [TFO]^−^, [BF_4_]^−^, Cl^−^, and the surface mainly comes from the interaction between the O, F, and Cl atoms of the anion and the P and As atoms on the surface of the phosphorene and arsenene. The distances between the cations and α-P, β-P, α-As, and β-As are 2.597~3.068 Å, 2.794~3.135 Å, 2.709~3.065 Å, and 2.848~2.963 Å, respectively. The distances between the anions and α-P, β-P, α-As, and β-As are 2.569~3.213 Å, 2.791~3.232 Å, 2.775~3.314 Å, and 2.899–3.133 Å, respectively. The above distances between the ILs and the nanosheets are much larger than the sum of the covalent radii and are close to the sum of the van der Waals radii of the corresponding atoms with the shortest distance. Therefore, we can preliminarily conclude that the ILs and 2D surfaces mainly combine via non-covalent interactions, as reported previously [28,29,30,31,32,33,34,35,36,38,39,40,41,42,43,44,45,46,47,48,49]. Furthermore, the configuration of the subject ILs underwent changes after adsorption. Despite the variation of the hydrogen bond distance between the cation and anion, the big difference is that the average position of the anion and cation is at a similar level from the surface after adsorption, while it is located above the imidazolium and pyridinium aromatic rings before adsorption. The change of the relative orientation between the anion and the cation of the ILs before and after adsorption results in the breaking of some old hydrogen bonds and the formation of new ones. In addition, we have also observed that the ionic liquid adsorption leads to a slight deformation of the nanosheets.

Adsorption energy is one of the criteria for evaluating interaction strength. As shown in Table 1, when the cations are the same, the order of the interaction between the same nanosheet and different ILs is: [X][Cl] > [X][TFO] > [X][BF_4_] (X = [EMIM]^+^, [MPI]^+^, [TMA]^+^). When the anions are the same, the order is: [EMIM][Y] ≈ [MPI][Y] > [TMA][Y] (Y = [TFO]^−^, [BF_4_]^−^, Cl^−^), which is due to the stronger interaction between the aromatic ring of the ILs and the nanosheets. More importantly, whether it is phosphorene or arsenene, the α-phase has a stronger interaction with ILs than does the β-phase. This may be due to the different structures of two phases (the puckered and buckled structures for the α- and β-phases). Obviously, the atomic density of the α-phase is larger than that of the β-phase. It can provide a more active contact site for ILs and consequently results in a strong interaction. Furthermore, the energy contributions from dispersion–correction are obtained from the difference of the corresponding adsorption energies computed at PBE and PBE-D2 levels (E_a_ vs. E_a_^D2^ in Table 1). It can be clearly seen that the vdW-correction makes a great contribution (ranging from 40%~80%) to the IL–nanosheet interaction. As presented in Table 1, for the system involving [X][TFO] and [X][BF_4_], the dispersion plays a decisive role in the adsorption process (70%~80%), while, for the system involving [X][Cl], the energy contribution from the dispersion–correction only amounts to 40%~50% because of the strongest adsorption affinity to the nanosheets. This further confirms the interaction between ILs and P or As nanosheets dominated by non-covalent forces and the importance of the dispersion–correction in describing these systems.

### 2.2. Valence Band Maximum, Conduction Band Minimum, and Density of States of IL/2D Sheets

The influence of IL adsorption on the electronic properties of subject 2D materials can be explored through the valence band maximum (VBM), conduction band minimum (CBM), and VBM–CBM gaps of the isolated nanosheets (α-P, β-P, α-As, and β-As), nine ILs, and IL/2D nanosheet hybrids, which are listed in Table 1. The energy gap of ILs lies between 1.28 and 6.01 eV. The calculated band gaps of α-P, β-P, α-As, and β-As are 0.53 eV, 1.93 eV, 0.47 eV, and 1.38 eV, respectively, which are similar to the previously reported PBE values [2,11]. Compared with that of pristine 2D materials, IL adsorption always results in the reduction of the band gap, and the extent of the decrease is closely related to the types of ILs and the surface. For α-P and α-As, the gap changes upon the adsorption of ILs, ranging from 0.00 to 0.08 eV, while there are relatively large changes for those of β-P and β-As (0.13~0.52 eV). In most cases, whether for phosphorene or arsenene, the IL adsorption has greater impact on the band gaps of the β-phase than that of the α-counterpart. If we further compare the effect of different, adsorbed ILs on the band gaps of β-P and β-As, the order is [X][Cl] > [X][TFO] > [X][BF_4_] (X = [EMIM]^+^, [MPI]^+^, [TMA]^+^). Moreover, the ILs including [MPI]^+^ also have a larger impact on the band gap of α- and β-nanosheets. The above results suggest that some of the considered ILs can be used to modulate the gaps of the phosphorene and arsenene, while others can preserve the electronic properties of the 2D materials (especially α-phase P and As).

**Figure 3:**
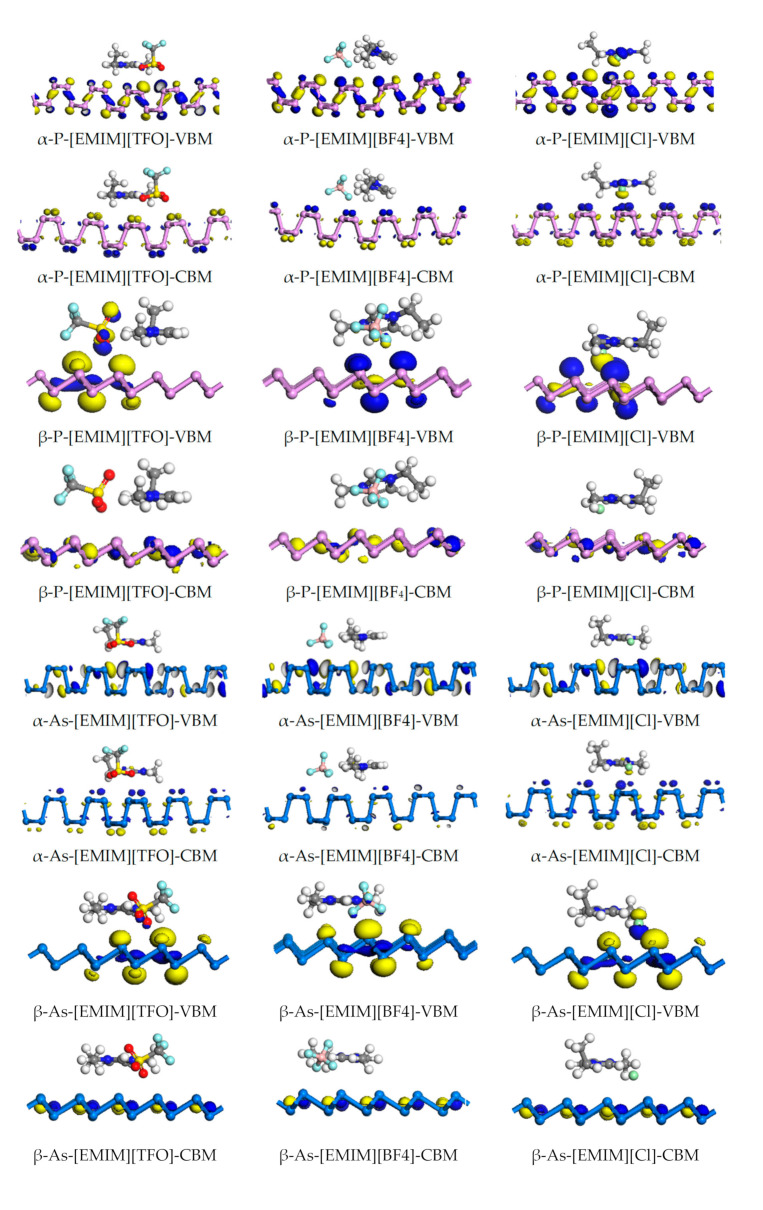
Electron density of CBM and VBM for complexes of [EMIM][TFO], [EMIM][BF_4_], and [EMIM][Cl] adsorbed on α-P and α-As at an isosurface value of 0.018 e/Å^3^, and β-P and β-As at an isosurface value of 0.030 e/Å^3^.

These band gap changes can be explained by the changes in VBM and CBM of the ILs/nanosheets adsorption system. For α-P and α-As, the effect of adsorption of [X][TFO] and [X][BF_4_] (X = [EMIM]^+^, [MPI]^+^, [TMA]^+^) on VBM and CBM is negligible. Although [X][Cl] adsorption has greater impact on the VBM and CBM of α-phase nanosheets than on other ionic liquids, the band shifts are almost the same. Therefore, the band gap of α-phase nanosheets is nearly unchanged. However, for nanosheets of β-P and β-As, the VBM state shifts to a higher level during [X][TFO] and [X][BF_4_] (X = [EMIM]^+^, [MPI]^+^, [TMA]_+_) adsorption, while the change of the CBM state is much smaller than that of VBM, resulting in a reduction in the overall band gap. [X][Cl] adsorption also has the largest impact on the VBM, CBM, and the gap (especially VBM) of β-phase nanosheets among all considered ionic liquids. In a word, the adsorption of ILs has greater influence on the band of β-phase nanosheets than on that of the α-phase counterparts. This clearly demonstrates that the IL adsorption effect on the electronic properties of phosphorene and arsenene are not only related to the chemical elements, but also closely related to the phase structures (α-phase: puckered and β-phase: buckled). The distinct behavior of α- and β-phase P and As upon IL adsorption can also be explored by their VBM and CBM electron density distributions in Figure 3. For the adsorption system involving α-P/As, the VBM and CBM are solely from the states of the surface, while for the system involving β-P/As, the relatively large contribution of the adsorbed ILs to the VBM can be observed.

**Figure 4:**
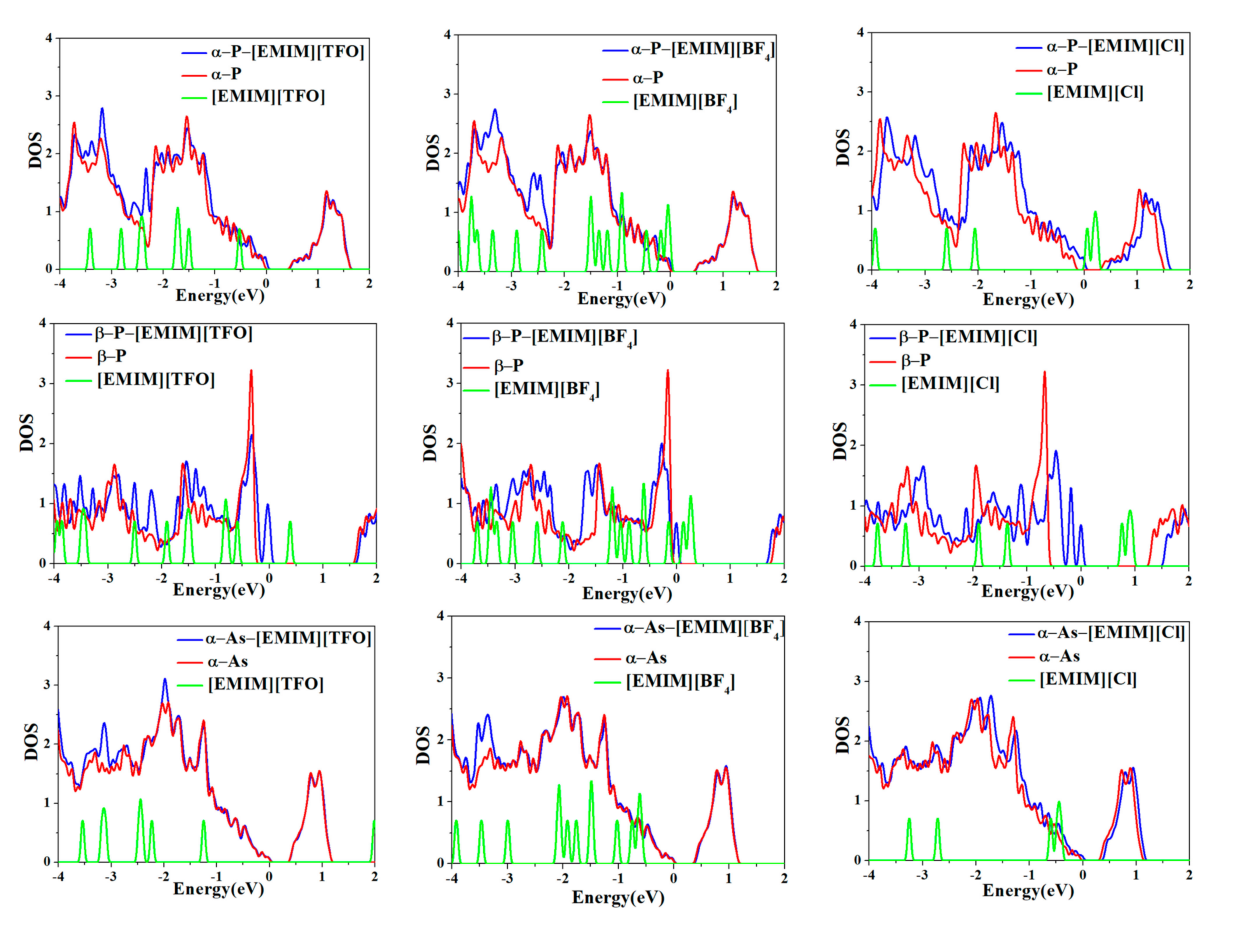

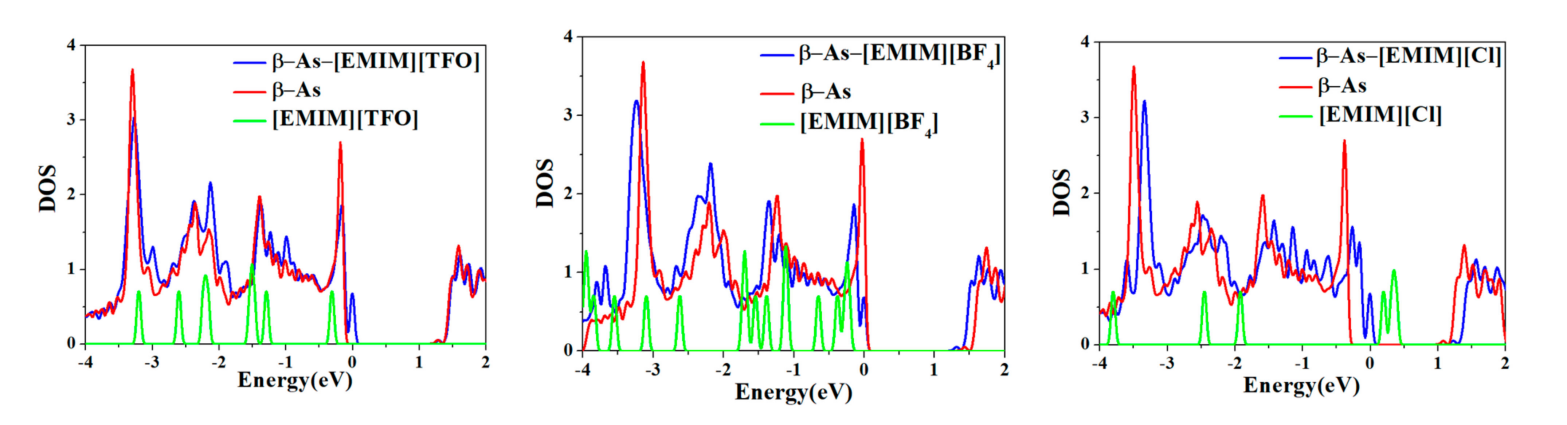
Density of states for pristine nanosheets (α-P, β-P, α-As, and β-As), isolated ILs ([EMIM][TFO], [EMIM][BF_4_], and [EMIM][Cl]), and adsorption systems of ILs on corresponding nanosheets.

To further examine the variations in the VBM/CBM gap caused by the IL adsorption, the density of states (DOS) for ILs, α-P/As, β-P/As, and ILs/surface complexes are illustrated in Figure 4 and Figure S4. As shown in the figures, in most cases, the shape and position of the DOS for P and As only change slightly after IL adsorption. Certainly, the above change is strongly dependent on the nature of the considered ILs and the nanosheets. Due to the non-covalent interaction between ILs and surfaces, the contribution of states near the Fermi level is mainly from phosphorene and arsenene. When comparing the adsorption effect on the DOS of α-phase and β-phase P and As, we can see the obvious change of the state near the Fermi level of the β-phase, which results in the band-gap reduction to a different extent, while, for the DOS of α-phase P and As, the change can be negligible in most cases. This also confirmed that the electronic properties of nanosheets with different phase structures exhibit different sensitivities to IL adsorption. Detailed information on the effect of different ILs is consistent with the discussion based on VBM and CBM data in Table 1. For [X][TFO] or [X][BF_4_], the adsorption has almost no effect on the DOS of α-phase P and As. For [X][Cl] on α-phase nanosheets, the adsorption effects on VBM and CBM states are roughly the same, resulting in the basically unchanged overall band gap, while, for the above ILs on β-P and β-As nanosheets the adsorption makes the VBM state shift to higher energy, and the shift of the CBM state is smaller, resulting in a decrease in the overall band gap. The above discussion further suggests that the electronic properties of α-P and α-As are less sensitive to IL adsorption than β-P and β-As.

### 2.3. Charge Transfer between Ionic Liquids and 2D Sheets

**Figure 5:**
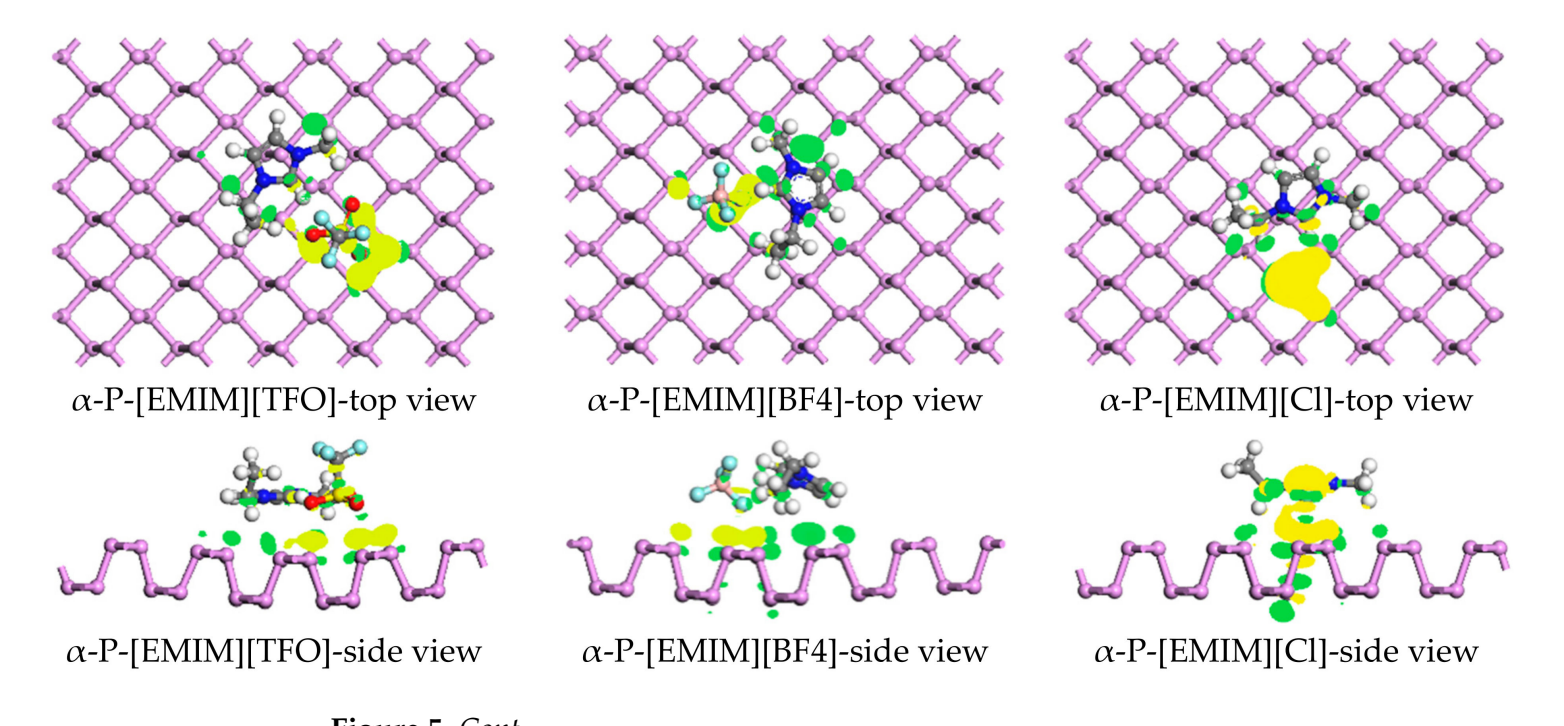

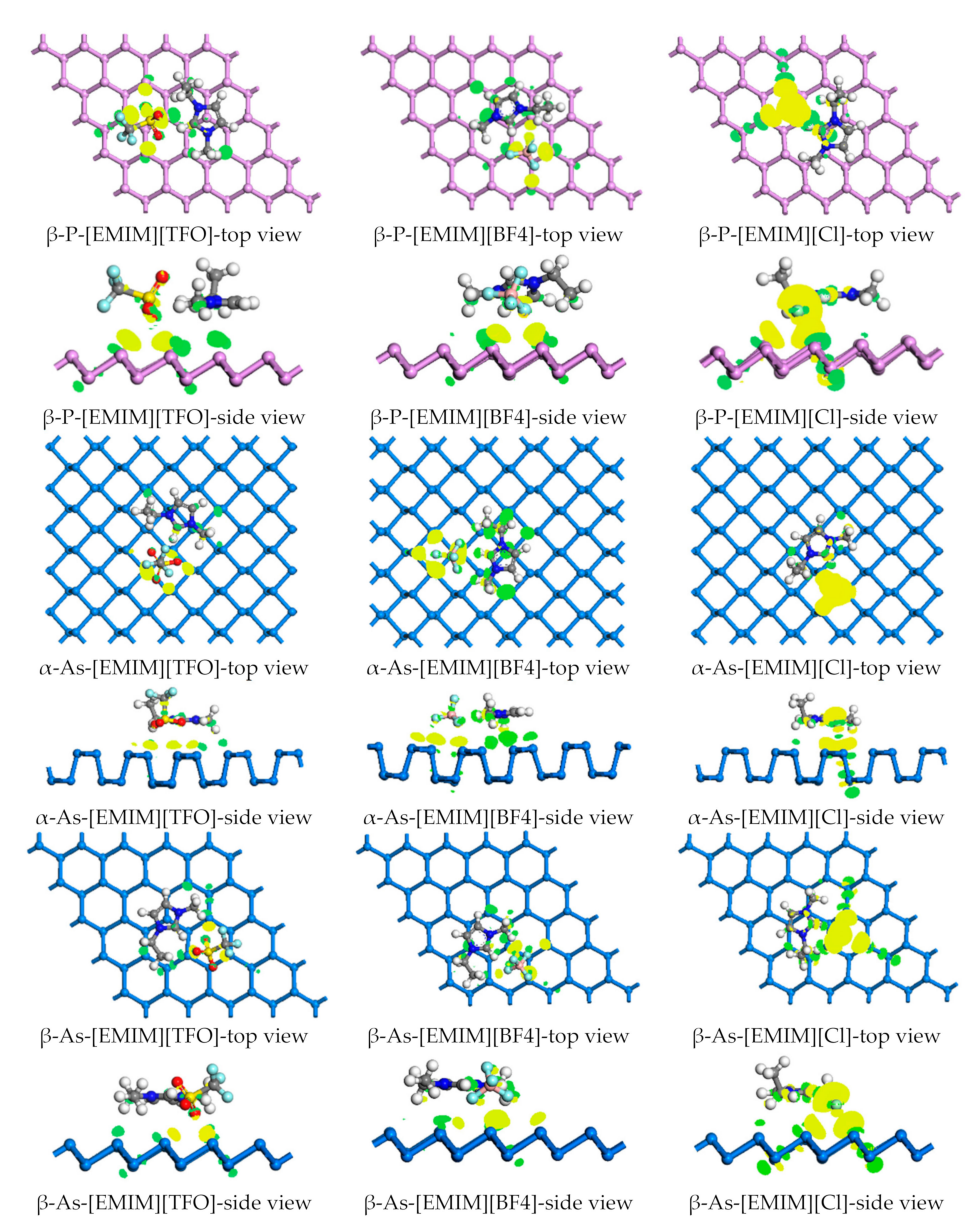
Top and side views of differential electron densities of ILs ([EMIM][TFO], [EMIM][BF_4_], and [EMIM][Cl]) adsorbed on α-P, β-P, α-As, and β-As nanosheets. Green and yellow areas correspond to accumulation and depletion of electronic densities, respectively, with an isosurface value of 0.003 e/Å^3^.

To further probe the interaction mechanism of the adsorption system, the differential charge density of ILs adsorbed on nanosheets in Figure 5 and Figure S5 can intuitively describe the electron redistribution upon adsorption. This parameter can be defined as:

Δρ = ρ_ILs/surface_ − ρ_surface_ − ρ_ILs._ (1)

where ρ_ILs/surface_, ρ_surface_, and ρ_ILs_ are the total electron density of the adsorption system, isolated nanosheets, and free ILs, respectively. The green and yellow areas represent the accumulation and depletion of electrons, which clearly show the electron density redistribution among the cation, anion, and nanosheet. The electron density is accumulated, and the surface is negatively charged underneath the cation, while it is opposite, underneath the anion. Obviously, the charge transfer between the [X][Cl] and the surface is larger than that of other systems involving [X][TFO] and [X][BF_4_]. A Mulliken population analysis can provide the quantitative information of the charge density difference of the adsorbed system. Table 1 shows the Mulliken charge redistribution of cations, anions, and surfaces after adsorption, which clearly suggest the electron transfer number and direction closely related to the types of ILs and nanosheets. A large amount of electron transfer occurred between the ILs containing [Cl]^−^ and the surfaces (up to 0.389 e), which is consistent with their strong adsorption strength and the large band gap reduction. For the purpose of comparison, the Hirshfeld charge difference of the ILs and nanosheets after adsorption is also shown in Table S2.

### 2.4. Optical Properties

The imaginary part of the dielectric function (*ε*_2_) is an effective parameter for measuring the light absorption of materials. It can be calculated using the CASTEP code, which is defined as:

(2) $$\varepsilon_{2}\left(q\to O_{\hat{u}}\;,\;\hbar \omega \right)=\frac{2e^{2}\pi }{\Omega \varepsilon_{0}}\underset{k,v,c}{\sum }{\left|\left\langle \psi_{k}^{c}\left|u\cdot r\right|\psi_{k}^{v}\right\rangle \right|}^{2}\delta \left(E_{k}^{c}-E_{k}^{v}-\hbar \omega \right)$$

where *u* is the vector defining the polarization of the incident electric field. This expression is similar to Fermi’s Golden Rule for time-dependent perturbations, and *ε*_2_(*ω*) can be thought of as detailing the real transitions between occupied and unoccupied electronic states.

**Figure 6:**
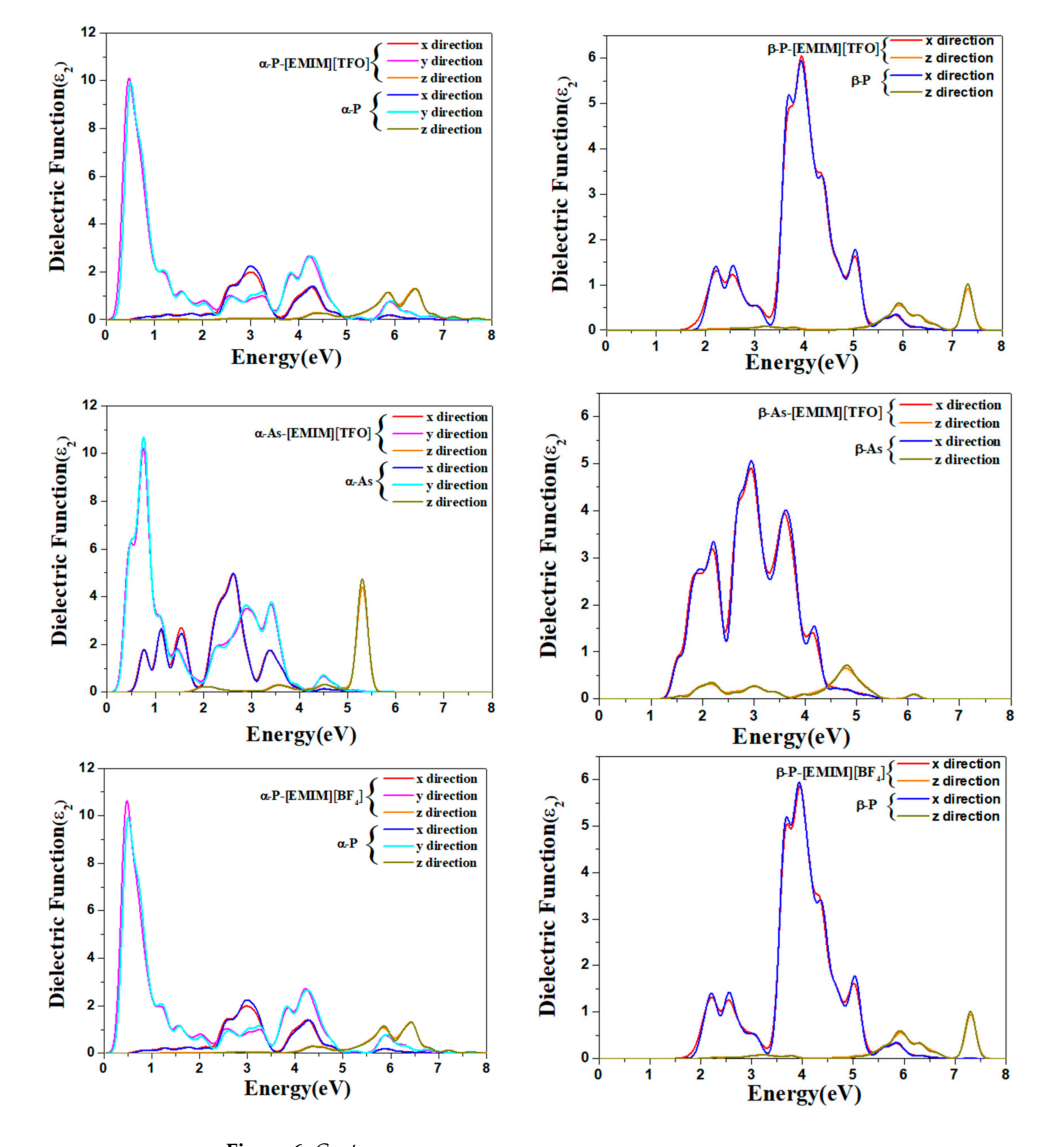

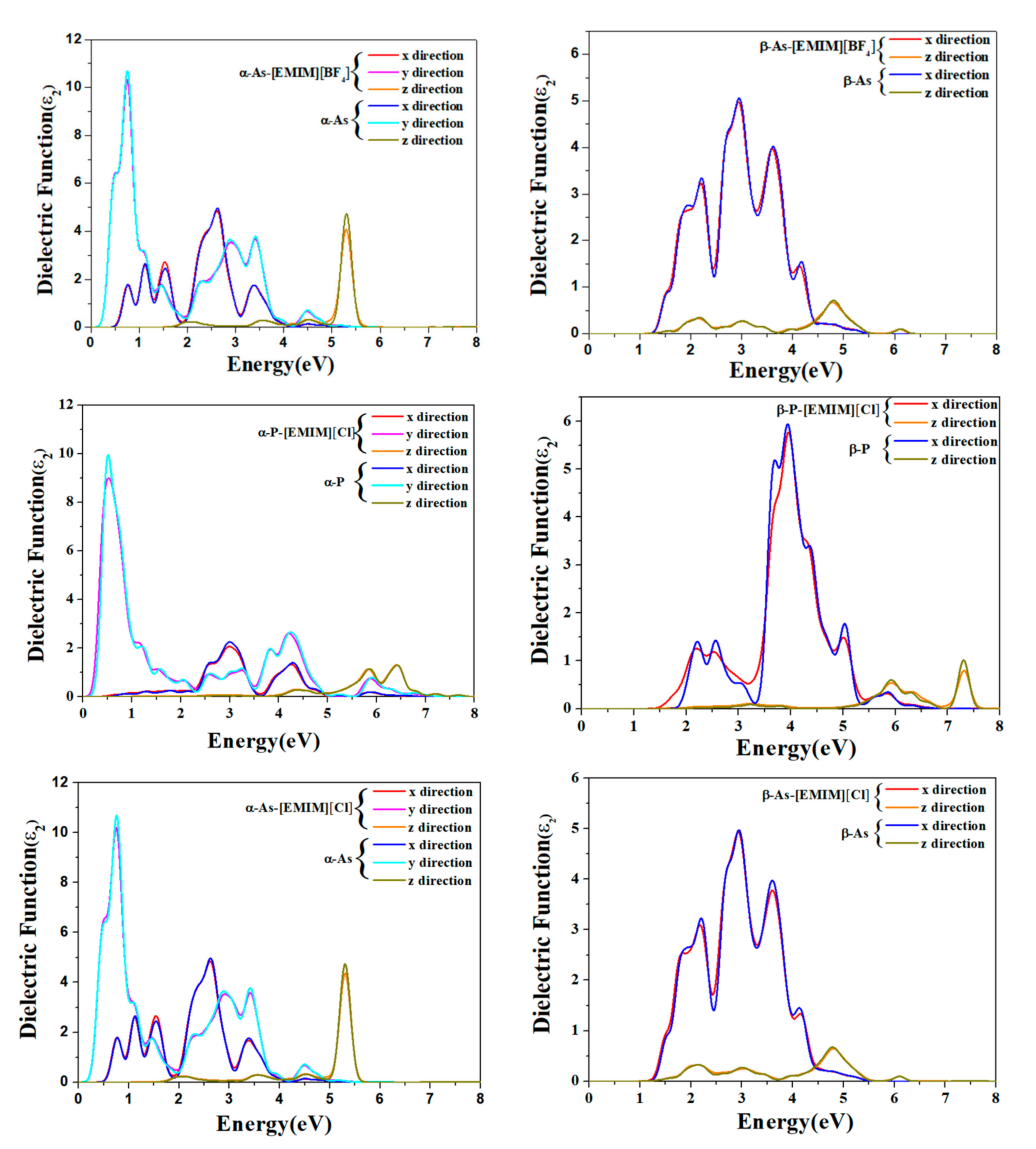
Computed, imaginary dielectric functions vs. energy for isolated and complexes of ILs ([EMIM][TFO], [EMIM][BF_4_], [EMIM][Cl]) adsorbed on α-P and α-As in the *x*, *y*, and *z* directions and β-P and β-As in the *x* and *z* direction.

The optical properties of the isolated and IL-adsorbed P and As nanosheets with α- and β-phases were calculated according to Equation (2). Figure 6 and Figure S6 show the *ε*_2_ along the three directions of the α-phase P and As. The *ε*_2_ for the β-phase nanosheets before and after adsorption are also shown along the two directions in the figures. Due to the in-plane anisotropy of α-phosphorene and α-arsenene along the *x* and *y* directions, the calculated *ε*_2_ has different characteristics in the two in-plane directions. However, β-phosphorene and β-arsenene exhibit the same characteristics in the *x* and *y* directions, so we only discuss the *x* direction in the β-phase nanosheets.

For α-P and α-As, the light polarized along the *x* and *y* directions shows a strong, wide range of light absorption starting from the energy of 0.5 eV and 0.2 eV. In addition, α-phosphorene and α-arsenene show strong ultraviolet absorption from 4 eV and 5 eV, respectively, along the z direction. As expected, for α-phase P and As, the IL adsorption has little effect on the peak position and light intensity of polarized light along the *x*, *y*, and *z* directions. For β-P, the light polarized along the *x* direction shows a strong, wide range of light absorption starting from the energy of 1.8 eV. The β-P nanosheets with adsorbed ILs along the *x* direction have a larger light absorption range of 1.5–1.8 eV than the isolated nanosheets. It can be observed that the absorption intensity and peak position shift in the *x* direction are more obvious after ILs adsorbed on the β-phase nanosheets, while there is only a slight change in the intensity of the ultraviolet light along the *z* direction. Therefore, the light absorption of the β-phase nanosheets is also more sensitive to ILs than are the α-phase counterparts.

## 3. Computational Details

The geometric optimization and electronic property computations were carried out using the DMol^3^ program [50]. The generalized gradient approximation (GGA) with the Perdew–Burke–Ernzerhof (PBE) function and the double numerical atomic basis set plus polarization (DNP) were employed [51]. Because of the poor description of the non-covalent interaction of the popular PBE function, an empirical dispersion-corrected density functional theory approach proposed by Grimme was used [52]. The performance of the DFT-D2 functional has been verified by López-Albarrán et al. based on their theoretical study of the adsorption mechanism of dibenzothiophene on boron-doped carbon nanoribbons [53]. The D values of the Grimme correction for DFT-D2 are shown in Table S1. The k-point was set to 5 × 5 × 1 for structural optimization and electronic property calculations. Electron density differences were computed by the CASTEP code.

To examine the adsorption behavior of ILs on the four subjected nanosheets (α-P, α-As, β-P, and β-As), 5 × 5 supercells containing 100 P or As atoms in the α phase and 50 P or As atoms in the β phase were used to simulate the 2D layers of P or As with different allotropes. The optimized lattice constants are a = 3.28 Å, b = 4.38 Å for α-P; a = 3.74 Å, b = 4.51 Å for α-As; and 3.33 Å and 3.79 Å for β-P and As nanosheets, respectively. The vacuum thickness was set to 20 Å to avoid artificial interactions between the periodic images. In line with previous work [28,29,30,31,32,33,34,35,36,38,39,40,41,42,43,44,45,46,47,48,49], the models for the adsorption system were constructed by placing a single ion pair of ILs on the surface of the investigational nanosheets.

To quantitatively describe the adsorption strength of ILs on P and As nanosheets, the adsorption energies (E_ad_) were calculated as:

E_ad_ = E_surface-IL_ − E_IL_ − E_surface_, (3)

where E_surface-IL_, E_IL_, and E_surface_ denote the energies of the surface-IL complex, the free ionic liquids, and the isolated nanosheets, respectively.

## 4. Conclusions

In this work, we investigated the adsorption characteristics of nine representative ILs based on the combinations of [EMIM]^+^, [MPI]^+^, and [TMA]^+^ cations paired to [BF_4_]^−^, [TFO]^−^, and Cl^−^ anions on phosphorene and arsenene with α- and β-phases. DFT calculations were performed to explore the effect of IL adsorption on the electronic and optical properties of the subject 2D materials. The calculated structure, adsorption energy, and charge transfer suggest that the interaction between ILs and P and As nanosheets is dominated by non-covalent forces, and the most stable adsorption structures are characterized by the simultaneous interaction of the cation and anion with the surface, irrespective of the types of ILs and surfaces. The discussion based on the VBM, CBM, and DOS of phosphorene and arsenene suggests that the adsorption of ILs has a much greater influence on the band of β-phase nanosheets than on that of the α-phase counterpart. In addition, the presence of ILs at the interface do not affect the optical properties of the α-phase P and As, while changes in some peak positions and absorption strengths are observed in the β-phase. This clearly demonstrates that the IL adsorption effect on the electronic properties of phosphorene and arsenene is not only related to the chemical elements, but also closely related to the phase structures. Through the thorough investigation of the nine-IL adsorption system, we can conclude that the electronic and optical properties of β-P and β-As are more sensitive to IL adsorption than are α-P and α-As. The above results also suggest that some of the considered ILs can be used to modulate the gaps of the phosphorene and arsenene, while others can preserve the electronic properties of the 2D materials (especially α-phase P and As). We hope that our calculated results can shed light on the effective screening and design of ionic liquid/2D materials to improve electrochemical-device performance, material dispersion, and surface catalytic activity.

## Figures and Tables

**Table 1:** Valence band maximum (VBM) and conduction band minimum (CBM) energies (in a.u.), corresponding gaps (Eg in eV), and gap changes before and after adsorption (ΔE in eV) of isolated 2D sheets, ionic liquids (ILs), and IL/2D sheet complexes for α-P, β-P, α-As, and β-As. Adsorption energies (Ea and Ea^D2^ at PBE and PBE-D2 level) of ILs on the surface. Charge difference of ionic liquids (ILs) and nanosheets after adsorption (values in e).

Structures	VBM	CBM	Eg(△E) (eV)	△q(+)^a^	△q(−)^b^	△q_sur_	E_a_ (eV)	E_a_^D2^ (eV)
EMIMTFO	−0.1990	−0.0799	3.24	−	−	−	−	−
EMIMBF_4_	−0.2497	−0.0763	4.72	−	−	−	−	−
EMIMCl	−0.1663	−0.0395	3.45	−	−	−	−	−
MPITFO	−0.1970	−0.1499	1.28	−	−	−	−	−
MPIBF_4_	−0.2435	−0.1372	2.89	−	−	−	−	−
MPICl	−0.1868	−0.0810	2.88	−	−	−	−	−
TMATFO	−0.1980	−0.0308	4.55	−	−	−	−	−
TMABF4	−0.2503	−0.0295	6.01	−	−	−	−	−
TMACl	−0.1521	−0.0108	3.84	−	−	−	−	−
α-P	−0.1819	−0.1624	0.53	−	−	−	−	−
β-P	−0.2241	−0.1533	1.93	−	−	−	−	−
α-As	−0.1552	−0.1378	0.47	−	−	−	−	−
β-As	−0.1936	−0.1431	1.38	−	−	−	−	−
α-P-EMIMTFO	−0.1796	−0.1619	0.48 (−0.05)	0.003	0.094	−0.095	0.30	1.20
β-P-EMIMTFO	−0.2132	−0.1515	1.68 (−0.25)	−0.016	0.096	−0.080	0.22	0.88
α-As-EMIMTFO	−0.1549	−0.1375	0.47 (0.00)	−0.040	0.045	−0.007	0.24	1.31
β-As-EMIMTFO	−0.1875	−0.1428	1.22 (−0.16)	−0.084	0.060	0.018	0.31	1.12
α-P-EMIMBF_4_	−0.1798	−0.1613	0.51 (−0.02)	0.001	0.073	−0.072	0.46	0.93
β-P-EMIMBF_4_	−0.2137	−0.1498	1.74 (−0.19)	−0.018	0.085	−0.067	0.37	0.83
α-As-EMIMBF_4_	−0.1549	−0.1371	0.48 (0.01)	−0.057	0.067	−0.012	0.31	1.18
β-As-EMIMBF_4_	−0.1892	−0.1434	1.24 (−0.14)	−0.078	0.043	0.032	0.37	1.04
α-P-EMIMCl	−0.1751	−0.1577	0.47 (−0.06)	0.252	0.108	−0.359	0.86	1.54
β-P-EMIMCl	−0.2008	−0.1434	1.56 (−0.37)	0.234	0.159	−0.389	0.89	1.51
α-As-EMIMCl	−0.1529	−0.1354	0.48 (0.01)	0.170	0.047	−0.214	0.69	1.60
β-As-EMIMCl	−0.1802	−0.1376	1.16 (−0.22)	0.160	0.090	−0.250	0.88	1.52
α-P-MPITFO	−0.1790	−0.1617	0.47 (−0.06)	−0.016	0.072	−0.058	0.23	1.12
β-P-MPITFO	−0.2117	−0.1516	1.64 (−0.29)	−0.014	0.110	−0.096	0.25	0.87
α-As-MPITFO	−0.1566	−0.1412	0.42 (−0.05)	−0.168	0.031	−0.139	0.14	1.12
β-As-MPITFO	−0.1889	−0.1465	1.15 (−0.23)	−0.103	0.037	0.065	0.25	1.04
α-P-MPIBF_4_	−0.1793	−0.1627	0.45 (−0.08)	−0.042	0.082	−0.041	0.29	1.10
β-P-MPIBF_4_	−0.2147	−0.1528	1.69 (−0.24)	−0.021	0.077	−0.057	0.17	0.72
α-As-MPIBF_4_	−0.1566	−0.1407	0.43 (−0.04)	−0.183	0.043	0.141	0.27	1.18
β-As-MPIBF_4_	−0.1892	−0.1471	1.15 (−0.23)	−0.134	0.061	0.074	0.32	0.99
α-P-MPICl	−0.1748	−0.1580	0.46 (−0.07)	0.358	−0.04	−0.323	0.63	1.39
β-P-MPICl	−0.2007	−0.1487	1.41 (−0.52)	0.392	−0.012	−0.384	0.59	1.17
α-As-MPICl	−0.1539	−0.1382	0.43 (−0.04)	0.161	−0.101	−0.064	0.52	1.43
β-As-MPICl	−0.1810	−0.1450	0.98 (−0.40)	0.270	−0.055	−0.214	0.54	1.22
α-P-TMATFO	−0.1781	−0.1603	0.49 (−0.04)	0.027	0.061	−0.083	0.35	0.98
β-P-TMATFO	−0.2107	−0.1483	1.70 (−0.23)	0.004	0.080	−0.083	0.24	0.74
α-As-TMATFO	−0.1539	−0.1364	0.48 (0.01)	−0.016	0.045	−0.029	0.25	1.03
β-As-TMATFO	−0.1850	−0.1405	1.21 (−0.17)	−0.051	0.054	−0.003	0.31	0.90
α-P-TMABF_4_	−0.1792	−0.1614	0.49 (−0.04)	0.006	0.065	−0.072	0.14	0.75
β-P-TMABF_4_	−0.2124	−0.1488	1.73 (−0.20)	−0.006	0.070	−0.062	0.15	0.62
α-As-TMABF_4_	−0.1545	−0.1369	0.48 (0.01)	−0.038	0.045	−0.005	0.18	0.89
β-As-TMABF_4_	−0.1875	−0.1416	1.25 (−0.13)	−0.053	0.038	0.015	0.24	0.79
α-P-TMACl	−0.1729	−0.1549	0.49 (−0.04)	0.149	0.202	−0.354	0.72	1.22
β-P-TMACl	−0.1963	−0.1398	1.54 (−0.39)	0.148	0.231	−0.380	0.70	1.13
α-As-TMACl	−0.1507	−0.1333	0.47 (0.00)	0.098	0.129	−0.227	0.48	1.10
β-As-TMACl	−0.1770	−0.1349	1.14 (−0.24)	0.088	0.146	−0.234	0.72	1.21

*△q(+)^a^ = q _cation in IL (after adsorption)_ − q _cation in IL (before adsorption)_; *△q(−)^b^ = q _anion in IL (after adsorption)_ − q _anion in IL (before adsorption)_.

## Data Availability

The data presented in this study are available in Supplementary Materials.

## Supplementary Materials

The following supporting information can be downloaded at: https://www.mdpi.com/article/10.3390/molecules27082518/s1, Table S1. The D values of Grimme correction for DFT-D2; Figure S1. The initial structures of different configurations of [EMIM][BF_4_] and [TMA][BF_4_] adsorbed on the 2D nanosheet; Figure S2. Most stable geometry of ionic liquid adsorbed on 2D surfaces with key interaction distances (Å); Figure S3. Electron density of CBM and VBM for complexes of [MPI][TFO], [MPI][BF_4_], [MPI][Cl], [TMA][TFO], [TMA][BF_4_], and [TMA][Cl] adsorbed on α-P and α-As at an isosurface value of 0.018 e/Å3, β-P and β-As at an isosurface value of 0.030 e/Å^3^; Figure S4. Density of states for pristine nanosheets (α-P, β-P, α-As, and β-As), isolated ionic liquids (ILs) ([MPI][TFO], [MPI][BF_4_], [MPI][Cl], [TMA][TMA], [TMA][BF_4_], and [TMA][Cl]), and adsorption systems of ILs on corresponding nanosheets; Figure S5. Top and side views of differential electron density of ILs ([MPI][TFO], [MPI][BF_4_], [MPI][Cl], [TMA][TFO], [TMA][BF_4_], and [TMA][Cl]) adsorbed on α-P, β-P, α-As, and β-As nanosheets. Green and yellow areas correspond to accumulation and depletion of electronic densities, respectively, with an isosurface value of 0.003 e/Å^3^; Figure S6. Computed imaginary dielectric functions vs. energy for isolated and complexes of ILs ([MPI][TFO], [MPI][BF_4_], [MPI][Cl], [TMA][TFO], [TMA][BF_4_], and [TMA][Cl]) adsorbed on α-P and α-As in the *x*, *y*, and *z* directions, and β-P and β-As in the *x* and *z* direction; and Table S2. The Mulliken and Hirshfeld charge difference of ionic liquids (ILs) and nanosheets after adsorption (values in e).

## References

[1] Tan C., Cao X., Wu X.J., He Q., Yang J., Zhang X., Chen J., Zhao W., Han S., Nam G.H. (2017). Recent Advances in Ultrathin Two-Dimensional Nanomaterials. Chem. Rev..

[2] Zhang S., Xie M., Li F., Yan Z., Li Y., Kan E., Liu W., Chen Z., Zeng H. (2016). Semiconducting Group 15 Monolayers: A Broad Range of Band Gaps and High Carrier Mobilities. Angew. Chem. Int. Ed. Engl..

[3] Zhang S., Yan Z., Li Y., Chen Z., Zeng H. (2015). Atomically Thin Arsenene and Antimonene: Semimetal-Semiconductor and Indirect-direct Band-gap Transitions. Angew. Chem. Int. Ed. Engl..

[4] Guo S.Y., Hu X.M., Zhou W.H., Liu X.H., Gao Y.J., Zhang S.L., Zhang K., Zhu Z., Zeng H.B. (2018). Mechanistic Understanding of Two-Dimensional Phosphorus, Arsenic, and Antimony High-Capacity Anodes for Fast-Charging Lithium/Sodium Ion Batteries. J. Phys. Chem. C.

[5] Kong X., Liu Q., Zhang C., Peng Z., Chen Q. (2017). Elemental Two-Dimensional nanosheets beyond graphene. Chem. Soc. Rev..

[6] Ambrosi A., Chua C.K., Bonanni A., Pumera M. (2014). Electrochemistry of Graphene and Related Materials. Chem. Rev..

[7] Yu X., Liang W.Y., Xing C.Y., Chen K.Q., Chen J.M., Huang W.C., Xie N., Qiu M., Yan X.B., Xie Z.J. (2020). Emerging 2D Pnictogens for catalytic applications: Status and Challenges. J. Mater. Chem. A.

[8] Huo C., Yan Z., Song X., Zeng H. (2015). 2D Materials via Liquid Exfoliation: A Review on Fabrication and Applications. Sci. Bull..

[9] Gui R.J., Jin H., Sun Y.J., Jiang X.W., Sun Z.J. (2019). Two-Dimensional Group-VA Nanomaterials beyong Black Phosphorus: Synthetic Mathods, Properties, Functional Nanostructures and Applications. J. Mater. Chem. A.

[10] Wu Z., Qi J., Wang W., Zeng Z., He Q. (2021). Emerging elemental two-dimensional materials for energy applications. J. Mater. Chem. A.

[11] Zhang S., Guo S., Chen Z., Wang Y., Gao H., Gomez-Herrero J., Ares P., Zamora F., Zhu Z., Zeng H. (2018). Recent Progress in 2D Group-VA Semiconductors: From Theory to Experiment. Chem. Soc. Rev..

[12] Jellett C., Plutnar J., Pumera M. (2020). Prospects for Functionalizing Elemental 2D Pnictogens: A Study of Molecular Models. ACS Nano.

[13] Esan F., Kecik D., Özçelik V.O., Kadioglu Y., Üzengi Aktürk O., Durgun E., Aktürk E., Ciraci S. (2019). Two-dimensional pnictogens: A review of recent progresses and future research directions. Appl. Phys. Rev..

[14] Walia S., Balendhran S., Ahmed T., Singh M., El-Badawi C., Brennan M.D., Weerathunge P. (2017). Ambient Protection of Few-Layer Black Phosphorus via Sequestration of Reactive Oxygen Species. Adv. Mater..

[15] Sui Y., Zhou J., Wang X., Wu L., Zhong S., Li Y. (2021). Recent Advances in Black-Phosphorus-Based Materials for electrochemical Energy Storage. Mater. Today.

[16] Zhu Z., Tomannek D. (2014). Semiconducting Layered Blue Phosphorus: A Computational Study. Phys. Rev. Lett..

[17] Zhang S., Zhang J., Zhang Y., Deng Y. (2017). Nanoconfined Ionic Liquids. Chem. Rev..

[18] Fedorov M.V., Kornyshev A.A. (2014). Ionic Liquids at Electrified Interfaces. Chem. Rev..

[19] Valencia H., Kohyama M., Tanaka S., Matsumoto H. (2009). Ab intio Study of EMIM-BF4 Crystal Interaction with a Li (100) surface as a Model for Ionic Liquid/Li Interfaces in Li-ion Batteries. J. Chem. Phys..

[20] Lahiri A., Borisenko N., Endres F. (2018). Electrochemical Synthesis of Battery Electrode Materials from Ionic Liquids. Top. Curr. Chem..

[21] Atilhan M., Altamash T., Aparicio S. (2019). Quantum Chemistry Insight into the Interactions Between Deep Eutectic Solvents and SO_2_. Molecules.

[22] Hayes R., Warr G.G., Atkin R. (2015). Structure and Nanostructure in Ionic Liquids. Chem. Rev..

[23] Chen M., Li S., Feng G. (2017). The Influence of Anion Shape on the Electrical Double Layer Microstructure and Capacitance of Ionic Liquids-Based Supercapacitors by Molecular Simulations. Molecules.

[24] Dong K., Liu X., Dong H., Zhang X., Zhang S. (2017). Multiscale Studies on Ionic Liquids. Chem. Rev..

[25] Watanabe M., Thomas M.L., Zhang S., Ueno K., Yasuda T., Dokko K. (2021). Application of Ionic Liquids to Energy Storage and Conversion Materials and Devices. Chem. Rev..

[26] Du L., Geng C., Zhang D., Lan Z., Liu C. (2016). Atomic Resolution Insights into the Structural Aggregations and Optical Properties of Neat Imidazolium-Based Ionic Liquids. J. Phys. Chem. B.

[27] Wang B., Qin L., Mu T., Xue Z., Gao G. (2017). Are Ionic Liquids Chemically Stable?. Chem. Rev..

[28] García G., Atilhan M., Aparicio S. (2014). Theoretical Study on the Solvation of C60 Fullerene by Ionic Liquids II: DFT Analysis of the Interaction Mechanism. J. Phys. Chem. B.

[29] Garcia G., Atilhan M., Aparicio S. (2015). Theoretical Study of Renewable Ionic Liquids in the Pure State and with Graphene and Carbon Nanotubes. J. Phys. Chem. B.

[30] Garcia G., Atilhan M., Aparicio S. (2015). Adsorption of Choline Benzoate Ionic Liquid on Graphene, Silicene, Germanene and Boron-Nitride Nanosheets: A DFT Perspective. Phys. Chem. Chem. Phys..

[31] Garcia G., Atilhan M., Aparicio S. (2016). In Silico Rational Design of Ionic Liquids for the Exfoliation and Dispersion of Boron Nitride Nanosheets. Phys. Chem. Chem. Phys..

[32] Bari R., Tamas G., Irin F., Aquino A.J.A., Green M.J., Quitevis E.L. (2014). Direct Exfoliation of Graphene in Ionic Liquids with aromatic groups. Colloid Surf. A-Physicochem. Eng. Asp..

[33] Shakourian-Fard M., Kamath G., Jamshidi Z. (2014). Trends in Physisorption of Ionic Liquids on Boron-Nitride Sheets. J. Phys. Chem. C.

[34] Shakourian-Fard M., Jamshidi Z., Bayat A., Kamath G. (2015). Meta-Hybrid Density Functional Theory Study of Adsorption of Imidazolium- and Ammonium-Based Ionic Liquids on Graphene Sheet. J. Phys. Chem. C.

[35] Shakourian-Fard M., Taimoory S.M., Semeniuchenko V., Kamath G., Trant J.F. (2018). The effect of ionic liquid adsorption on the electronic and optical properties of flfluorographene nanosheets. J. Mol. Liq..

[36] Shakourian-Fard M., Ghenaatian H.R., Kamath G., Taimoory S.M. (2020). Unraveling the effect of nitrogen doping on graphene nanoflflakes and the adsorption properties of ionic liquids: A DFT study. J. Mol. Liq..

[37] Zhao W., Xue Z., Wang J., Jiang J., Zhao X., Mu T. (2015). Large-Scale, Highly Efficient, and Green Liquid-Exfoliation of Black Phosphorus in Ionic Liquids. ACS Appl. Mater. Interfaces.

[38] Herrera C., Alcalde R., Atilhan M., Aparicio S. (2014). Theoretical Study on Amino Acid-Based Ionic Pairs and Their Interaction with Carbon Nanostructures. J. Phys. Chem. C.

[39] Ruzanov A., Lembinen M., Ers H., García de la Vega J.M., Lage-Estebanez I., Lust E., Ivanistsev V.B. (2018). Density Functional Theory Study of Ionic Liquid Adsorption on Circumcoronene Shaped Graphene. J. Phys. Chem. C.

[40] He Y., Guo Y., Yan F., Yu T., Liu L., Zhang X., Zheng T. (2021). Density functional theory study of adsorption of ionic liquids on graphene oxide surface. Chem. Eng. Sci..

[41] Ghatee M.H., Moosavi F. (2011). Physisorption of Hydrophobic and Hydrophilic 1-Alkyl-3-methylimidazolium Ionic Liquids on the Graphenes. J. Phys. Chem. C.

[42] Lalitha M., Lakshmipathi S. (2017). Interface energetics of [Emim]^+^[X]^−^ and [Bmim]^+^[X]^−^ (X = BF_4_, Cl, PF_6_, TfO, Tf_2_N) based ionic liquids on graphene, defective graphene, and graphyne surfaces. J. Mol. Liq..

[43] Talaei R., Khalili B., Mokhtary M. (2020). Modulation of opto-electronic properties of the functionalized hexagonal boron nitride nanosheets with tunable aryl alkyl ionic liquids (TAAILs): Defect based analysis. J. Mol. Liq..

[44] Zhour K., Otero-Mato J.M., Hassan F.E.H., Fahs H., Vaezzadeh M., López-Lago E., Gallego L.J., Varela L.M. (2020). Electronic and optical properties of borophene and graphene with an adsorbed ionic liquid: A density functional theory study. J. Mol. Liq..

[45] Lu Y., Xu Y., Lu L., Xu Z., Liu H. (2021). Interfacial interactions and structures of protic ionic liquids on a graphite surface: A first-principles study and comparison with aprotic ionic liquids. Phys. Chem. Chem. Phys..

[46] Anderson E., Grozovski V., Siinor L., Siimenson C., Ivaništšev V., Lust K., Kallip S., Lust E. (2013). Influence of the electrode potential and in situ STM scanning conditionson the phase boundary structure of the single crystal Bi(111)|1-butyl-4-methylpyridinium tetrafluoroborate interface. J. Electroanal. Chem..

[47] Siinor L., Siimenson C., Ivaništšev V., Lust K., Lust E. (2012). Influence of cation chemical composition and structure on the double layer capacitance for Bi(111)|room temperature ionic liquid interface. J. Electroanal. Chem..

[48] Lu Y., Hong Y., Xu Z., Liu H. (2021). Interfacial interactions and structures of imidazolium-based ionic liquids on black phosphorus surface from first-principles. J. Mol. Liq..

[49] Tang Y., Huai W., Li H., Mao X., Xie J., Lee J.Y., Fu A. (2021). Adsorption of [BF_4_]^−^ Anion-Based Ionic Liquids on Phosphorene, Arsenene, and Antimonene: A Density Functional Theory Study. Int. J. Quantum Chem..

[50] Delley B. (2000). From molecules to solids with the DMol3 approach. Chem. Phys..

[51] Perdew J.P., Burke K., Ernzerhof M. (1996). Generalized Gradient Approximation Made Simple. Phys. Rev. Lett..

[52] Grimme S. (2006). Semiempirical GGA-type density functional constructed with a long-range dispersion correction. J. Comput. Chem..

[53] López-Albarrán P., Navarro-Santos P., Garcia-Ramirez M.A., Ricardo-Chávez J.L. (2015). Dibenzothiophene adsorption at boron doped carbon nanoribbons studied within density functional theory. J. Appl. Phys..

