# Supplementary material for: The Influence of Ionic Liquids Adsorption on the Electronic and Optical Properties of Phosphorene and Arsenene with Different Phases: A Computational Study
Source: Molecules. 2022 Apr 13;27(8):2518. doi: 10.3390/molecules27082518 (PMC9027769; doi:10.3390/molecules27082518)
Supplement: Supplementary file 1 [file molecules-27-02518-s001.zip › molecules-1670131-supplementary.pdf]

Supplementary Information

Contents

Table S1. The D values of Grimme correction for DFT-D2.

Figure S1. The initial structures of different configurations of [EMIM][BF4] and [TMA][BF4] adsorbed on the 2D nanosheet.

Figure S2. Most stable geometry of ionic liquid adsorbed on 2D surfaces with key interaction distances (Å).

Figure S3. Electron density of CBM and VBM for complexes of [MPI][TFO], [MPI][BF_4], [MPI][Cl], [TMA][TFO], [TMA][BF_4], and [TMA][Cl] adsorbed on α-P and α-As at a isosurface value of 0.018e/$Å^3$, β-P and β-As at a isosurface value of 0.030e/$Å^3$.

Figure S4. Density of states for pristine nanosheets (α-P, β-P, α-As, β-As), isolated ILs ([MPI][TFO], [MPI][BF_4], [MPI][Cl], [TMA][TMA], [TMA][BF_4], and [TMA][Cl]), and adsorption systems of ILs on corresponding nanosheets.

Figure S5. Top and side views of differential electron density of ILs([MPI][TFO], [MPI][BF4], [MPI][Cl], [TMA][TFO], [TMA][BF_4], and[TMA][Cl]) adsorbed on α-P, β-P, α-As, and β-As nanosheets. Green and yellow areas correspond to accumulation and depletion of electronic densities, respectively, with an isosurface value of 0.003e/$Å^3$.

Figure S6. Computed imaginary dielectric functions versus energy for isolated and complexes of ILs ([MPI][TFO], [MPI][BF_4], [MPI][Cl], [TMA][TFO], [TMA][BF_4], and [TMA][Cl]) adsorbed on α-P and α-As in the x, y, z direction, and β-P and β-As in the x, z direction.

Table S2. The Mulliken and Hirshfeld charge difference of ionic liquids (ILs) and nanosheets after adsorption (values in e).

Table S 1. The D values of Grimme correction for DFT-D2.

Element	C_6 (eV Å ^{6})	R_0 (Å)
H	1.4510	1.0010
B	32.4402	1.4850
C	18.1375	1.4520
N	12.7481	1.3970
O	7.2550	1.3420
F	7.7732	1.2870
S	57.7290	1.6830
Cl	52.5468	1.6390
P	81.2559	1.7050
As	169.6631	1.7600

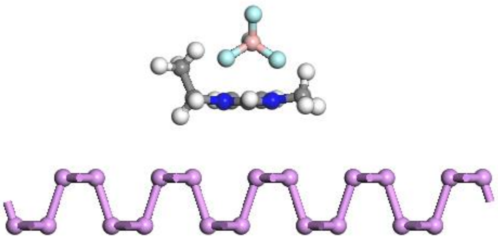

α-P-[EMIM][BF_4]-1

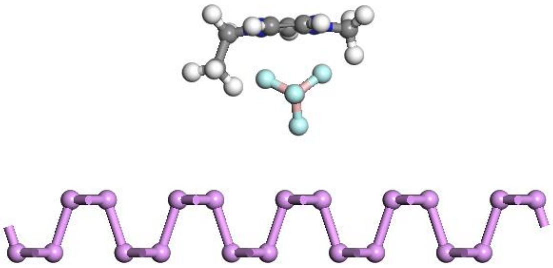

α-P-[EMIM][BF_4]-2

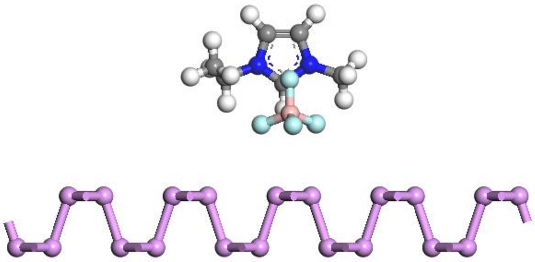

α-P-[EMIM][BF_4]-3

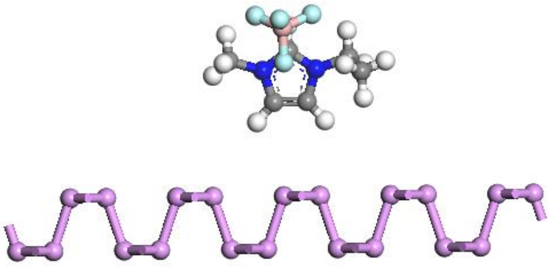

α-P-[EMIM][BF_4]-4

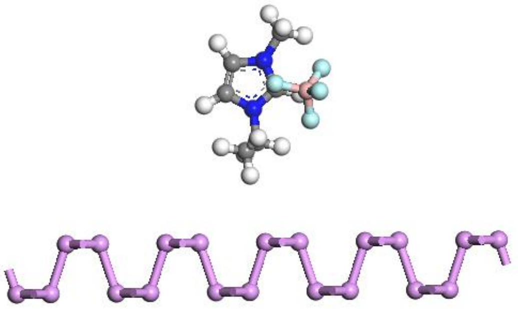

α-P-[EMIM][BF_4]-5

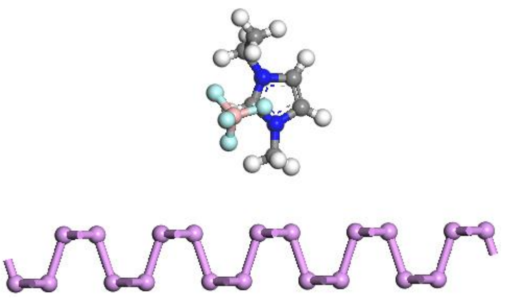

α-P-[EMIM][BF_4]-6

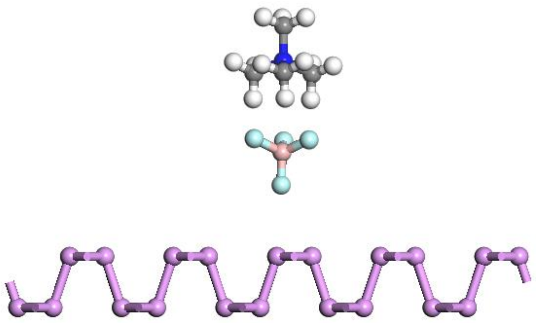

α-P-[TMA][BF_4]-1

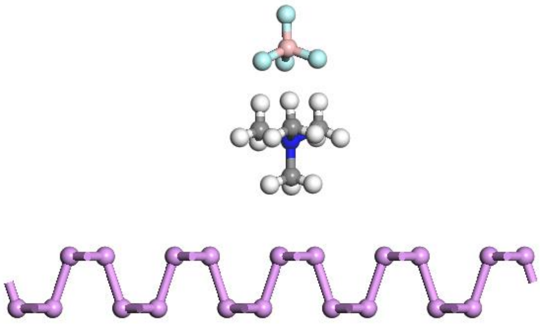

α-P-[TMA][BF_4]-2

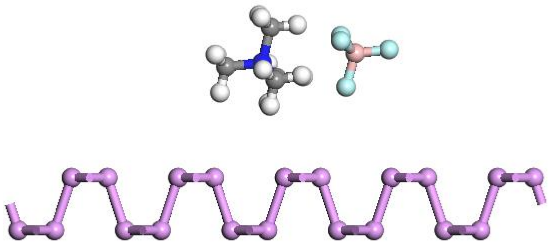

α-P-[TMA][BF_4]-3

Figure S1. The initial structures of different configurations of [EMIM][BF4] and [TMA][BF4] adsorbed on the 2D nanosheet.

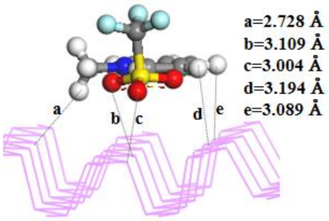

α-P-[MPI][TFO]

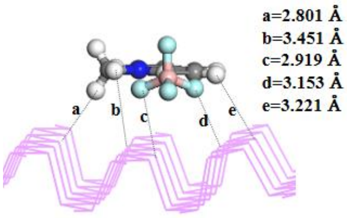

α-P-[MPI][BF_4]

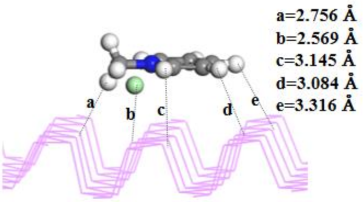

α-P-[MPI][Cl]

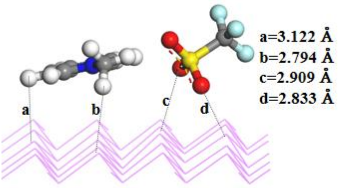

β-P-[MPI][TFO]

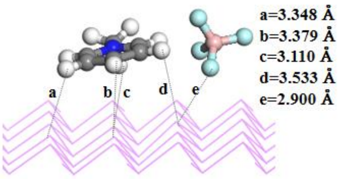

β-P-[MPI][BF_4]

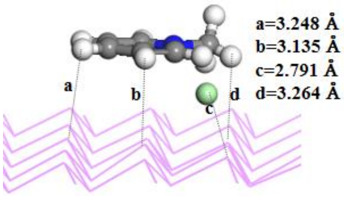

β-P-[MPI][Cl]

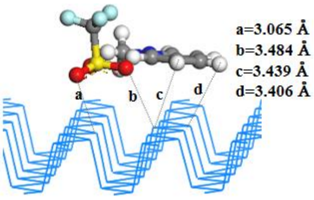

α-As-[MPI][TFO]

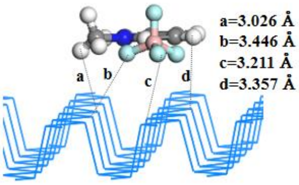

α-As-[MPI][BF_4]

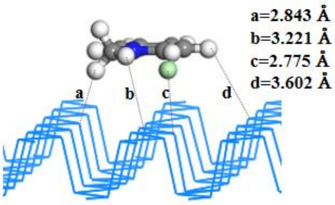

α-As-[MPI][Cl]

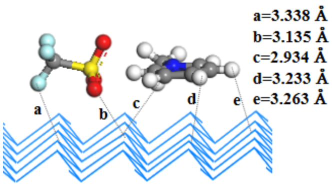

β-As-[MPI][TFO]

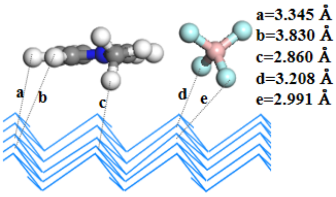

β-As-[MPI][BF_4]

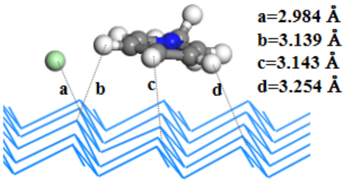

β-As-[MPI][Cl]

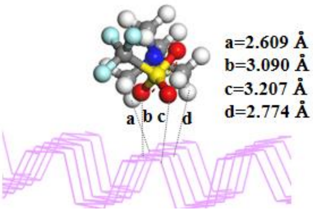

α-P-[TMA][TFO]

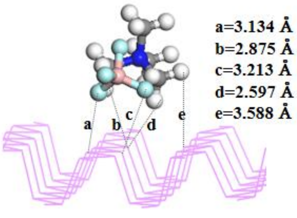

α-P-[TMA][BF_4]

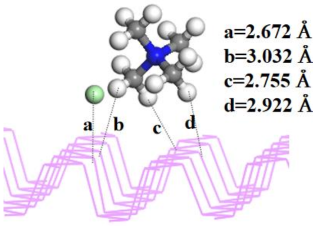

α-P-[TMA][Cl]

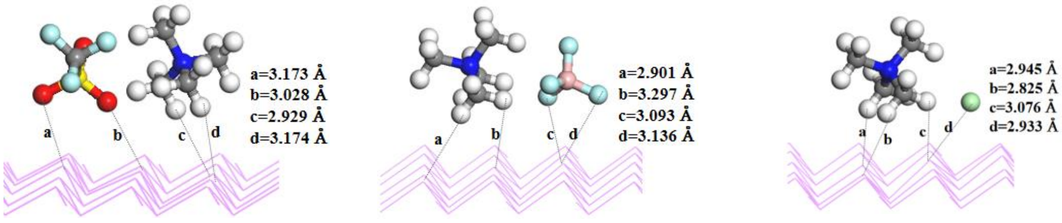

β-P-[TMA][TFO] β-P-[TMA][BF_4] β-P-[TMA][Cl]

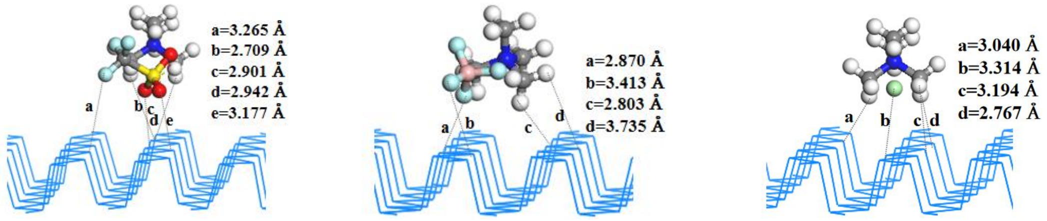

α-As-[MPI][TFO] α-As-[TMA][BF_4] α-As-[TMA][Cl]

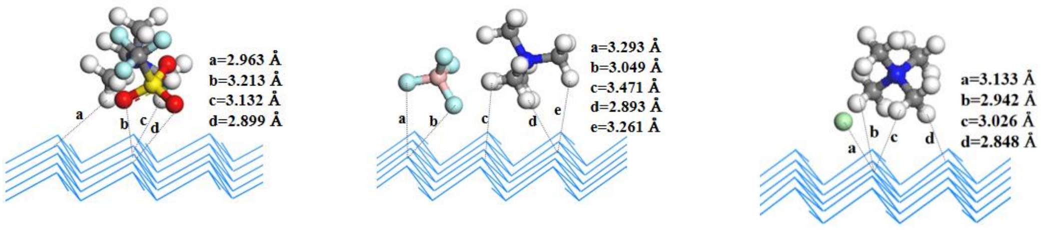

β-As-[TMA][TFO] β-As-[TMA][BF_4] β-As-[TMA][Cl]

Figure S2. Most stable geometry of ionic liquid adsorbed on 2D surfaces with key interaction distances (Å).

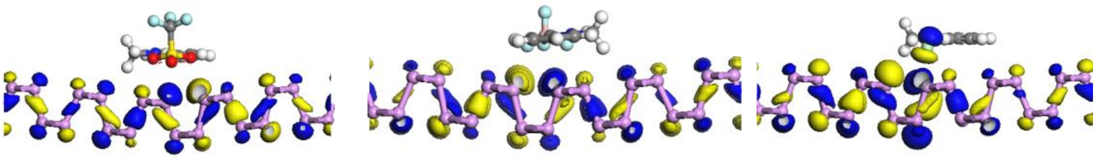

α-P-[MPI][TFO]-VBM α-P-[MPI][BF_4]-VBM α-P-[MPI][Cl]-VBM

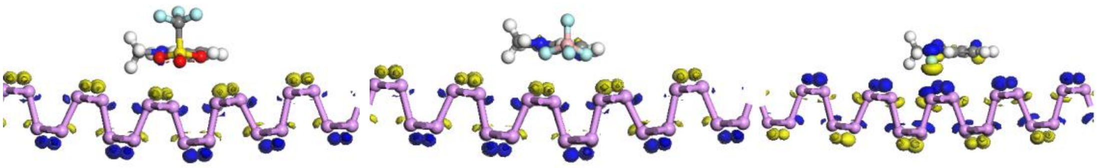

α-P-[MPI][TFO]-CBM α-P-[MPI][BF_4]-CBM α-P-[MPI][Cl]-CBM

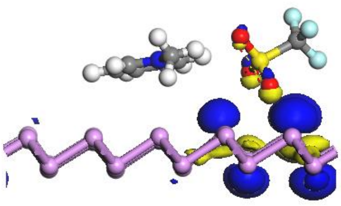
β-P-[MPI][TFO]-VBM

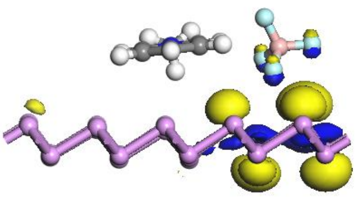
β-P-[MPI][BF_4]-VBM

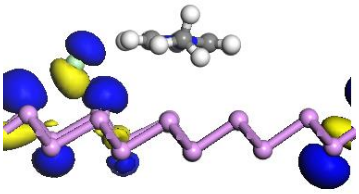
β-P-[MPI][Cl]-VBM

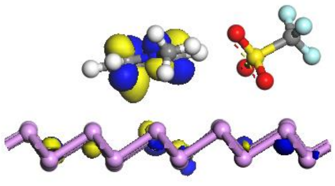
β-P-[MPI][TFO]-CBM

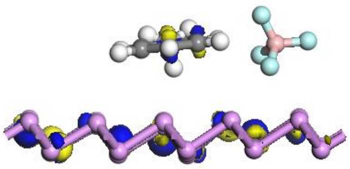
β-P-[MPI][BF_4]-CBM

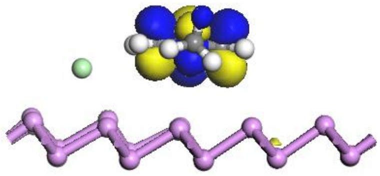
β-P-[MPI][Cl]-CBM

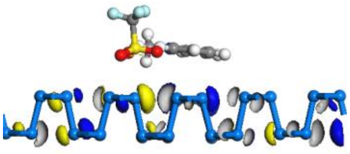
α-As-[MPI][TFO]-VBM

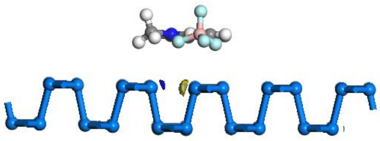
α-As-[MPI][BF_4]-VBM

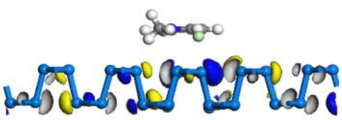
α-As-[MPI][Cl]-VBM

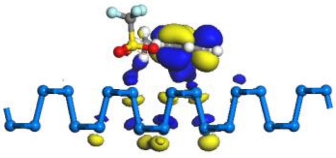
α-As-[MPI][TFO]-CBM

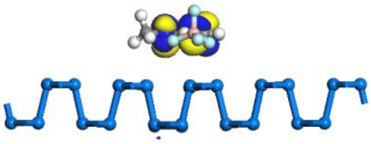
α-As-[MPI][BF_4]-CBM

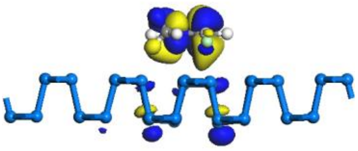
α-As-[MPI][Cl]-CBM

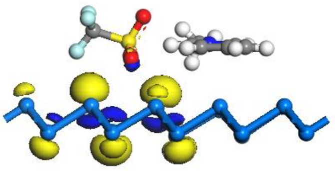
β-As-[MPI][TFO]-VBM

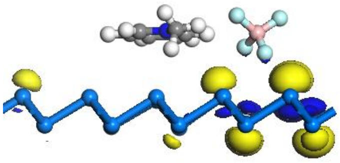
β-As-[MPI][BF_4]-VBM

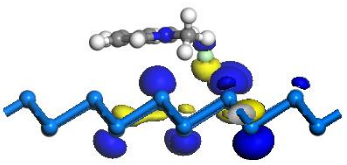
β-As-[MPI][Cl]-VBM

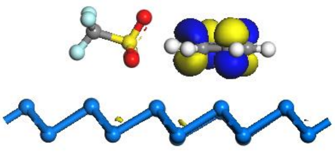
β-As-[MPI][TFO]-CBM

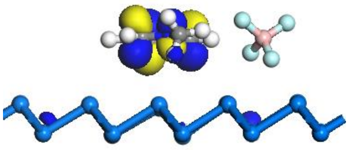
β-As-[MPI][BF_4]-CBM

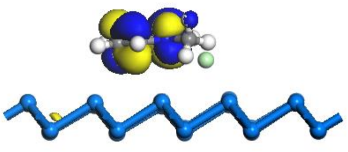
β-As-[MPI][Cl]-CBM

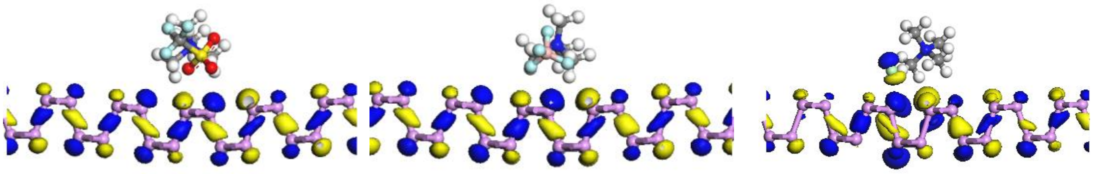

α-P-[TMA][TFO]-VBM α-P-[TMA][BF_4]-VBM α-P-[TMA][Cl]-VBM

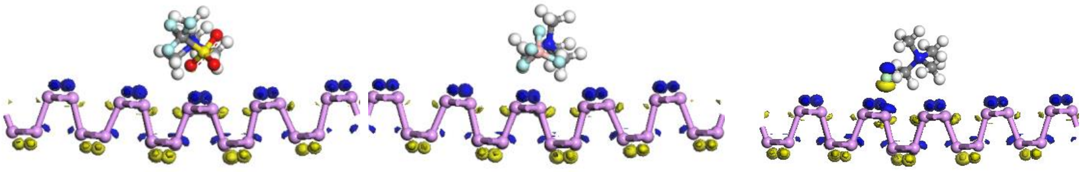

α-P-[TMA][TFO]-CBM α-P-[TMA][BF_4]-CBM α-P-[TMA][Cl]-CBM

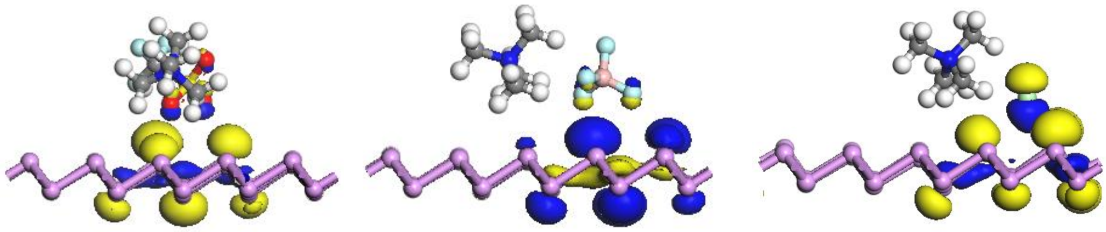

β-P-[TMA][TFO]-VBM β-P-[TMA][BF_4]-VBM β-P-[TMA][Cl]-VBM

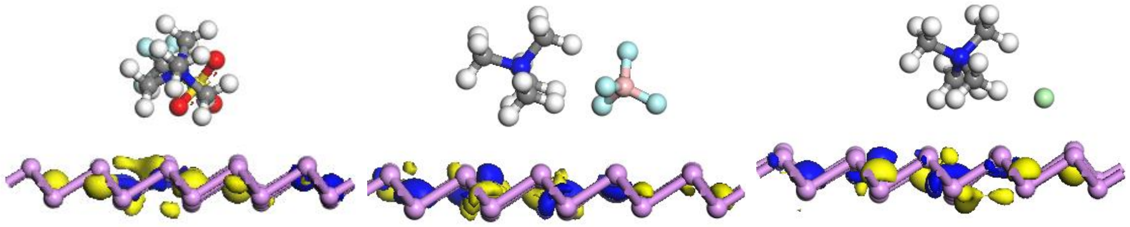

β-P-[TMA][BF_4]-CBM β-P-[TMA][BF_4]-CBM β-P-[TMA][Cl]-CBM

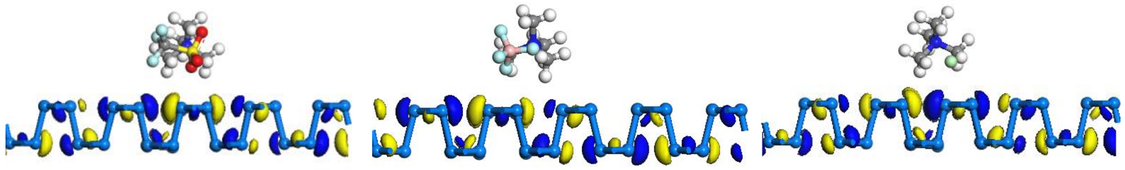

α-As-[TMA][TFO]-VBM α-As-[TMA][BF_4]-VBM α-As-[TMA][Cl]-VBM

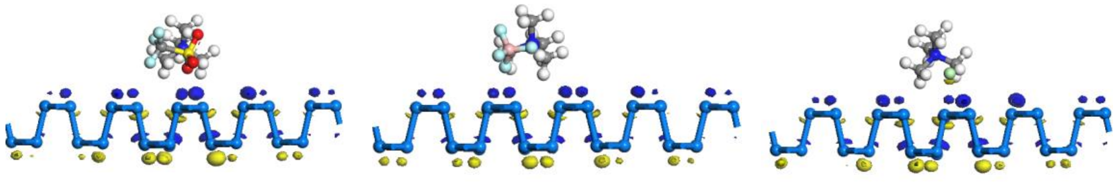

α-As-[TMA][TFO]-CBM α-As-[TMA][BF_4]-CBM α-As-[TMA][Cl]-CBM

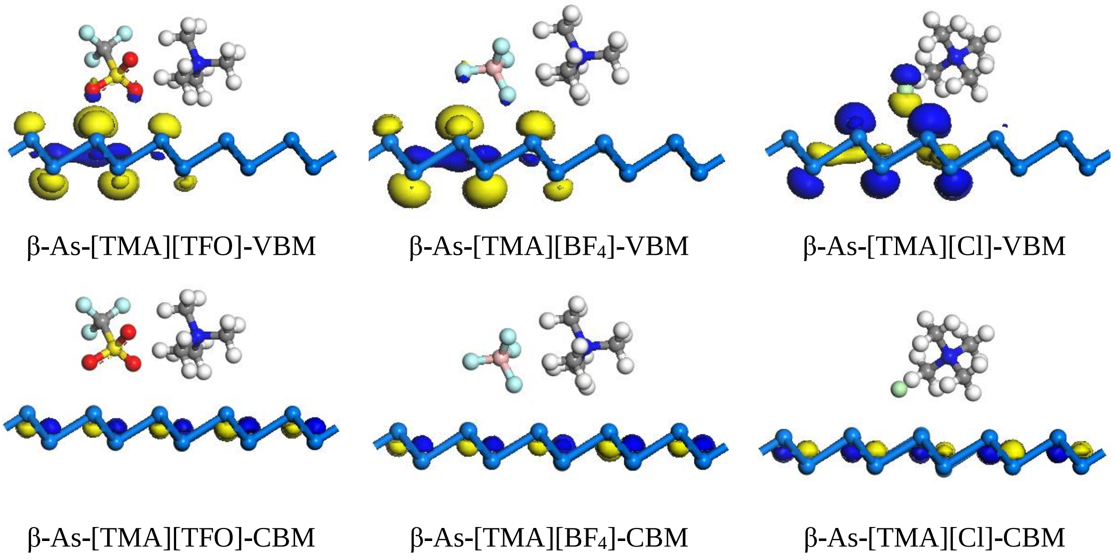

Figure S3. Electron density of CBM and VBM for complexes of [MPI][TFO], [MPI][BF_4], [MPI][Cl], [TMA][TFO], [TMA][BF_4], and [TMA][Cl] adsorbed on α-P and α-As at a isosurface value of 0.018e/Å^3, β-P and β-As at a isosurface value of 0.030e/Å^3

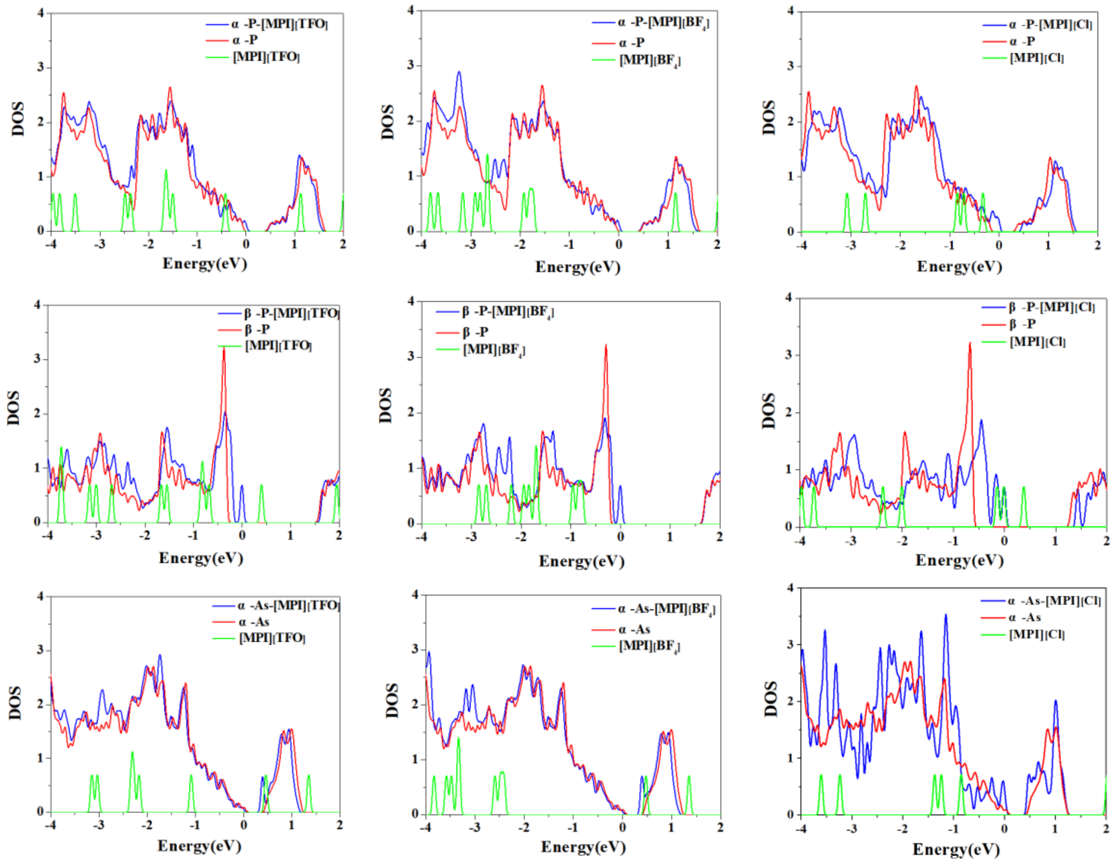

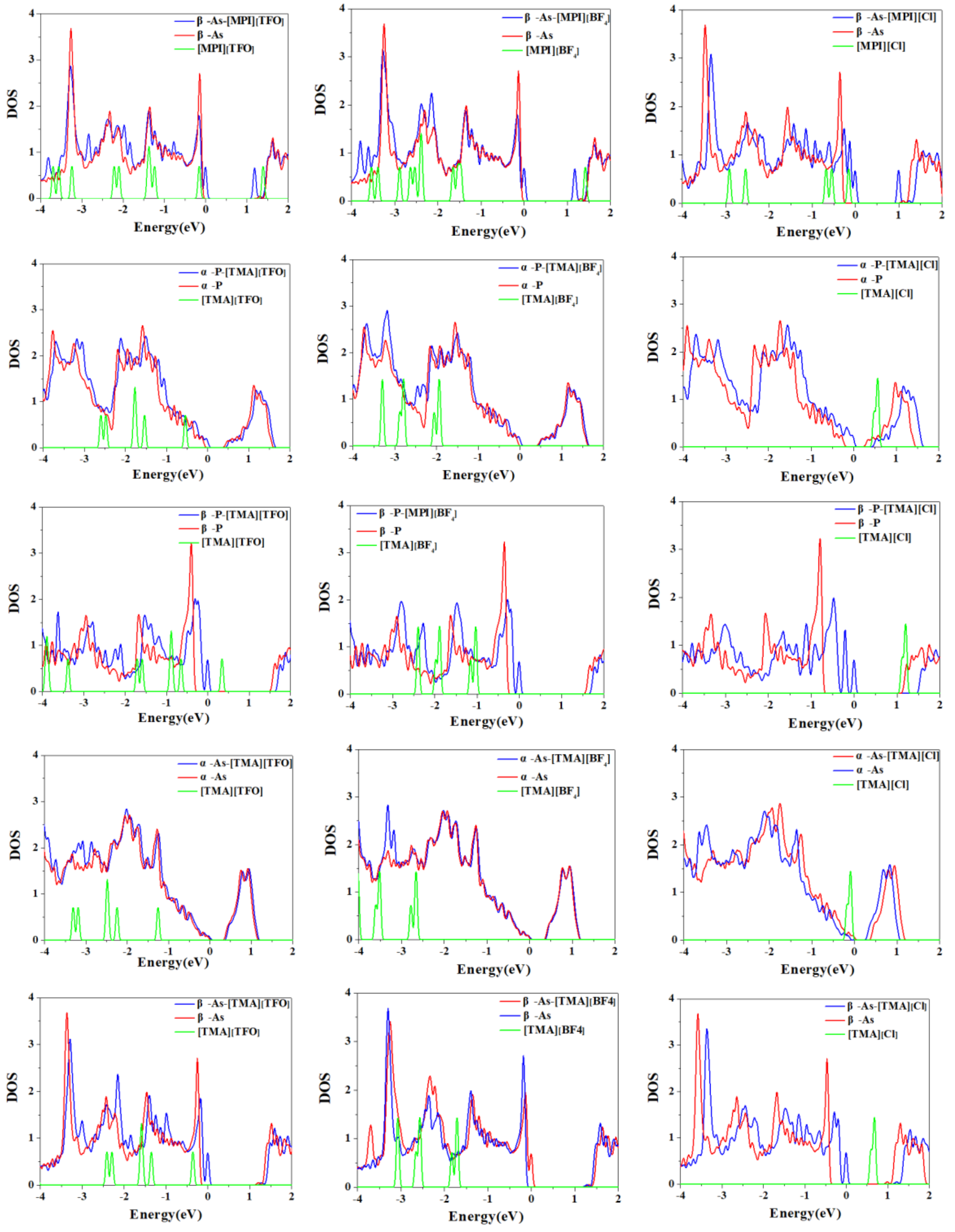

Figure S4. Density of states for pristine nanosheets (α-P, β-P, α-As, β-As), isolated ILs ([MPI][TFO], [MPI][BF_4], [MPI][Cl], [TMA][TMA], [TMA][BF_4], and [TMA][Cl]), and adsorption systems of ILs on corresponding nanosheets

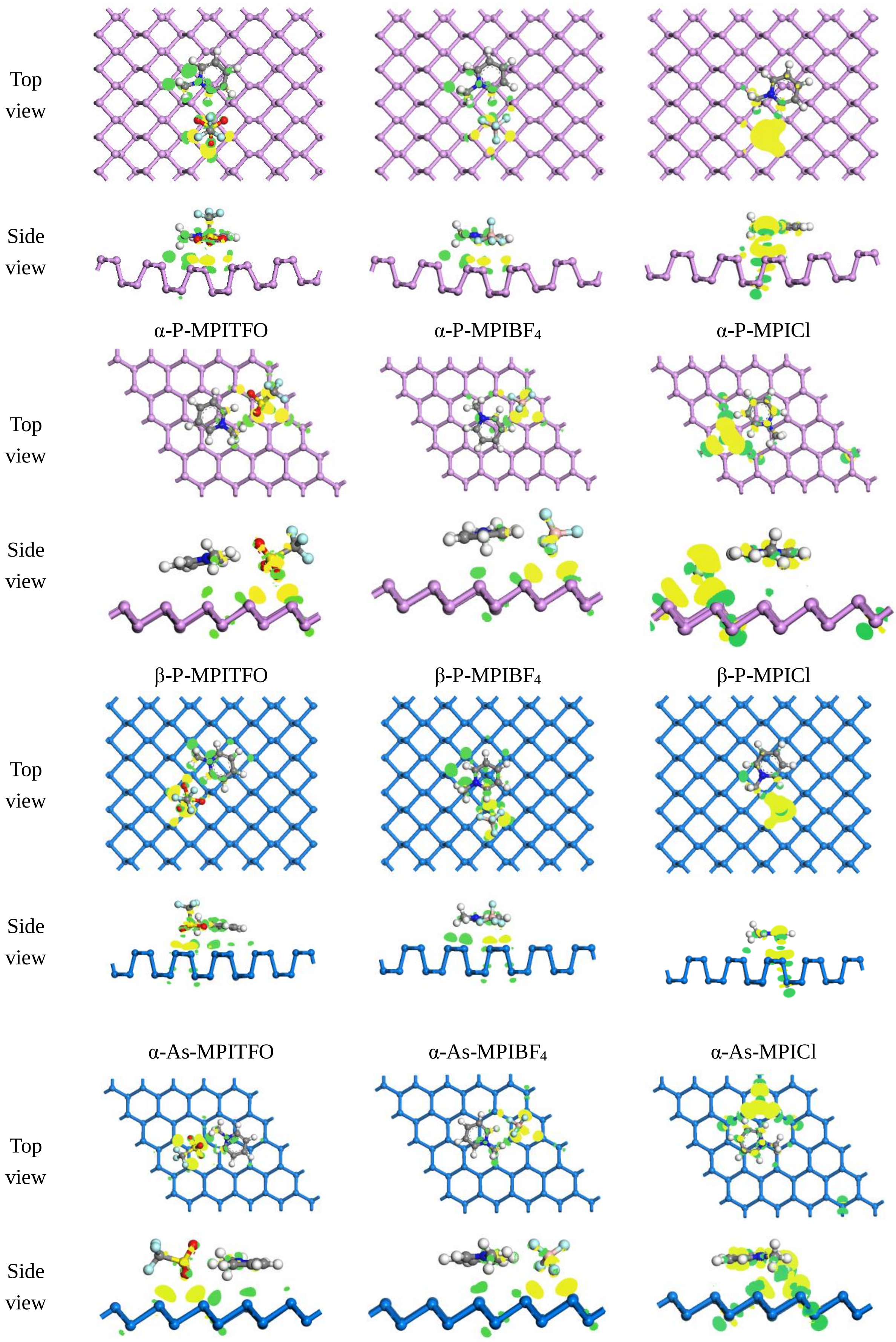

Top view
Side view
α-P-MPITFO
α-P-MPIBF$_4$
α-P-MPICl
Top view
Side view
β-P-MPITFO
β-P-MPIBF$_4$
β-P-MPICl
Top view
Side view
α-As-MPITFO
α-As-MPIBF$_4$
α-As-MPICl
Top view
Side view

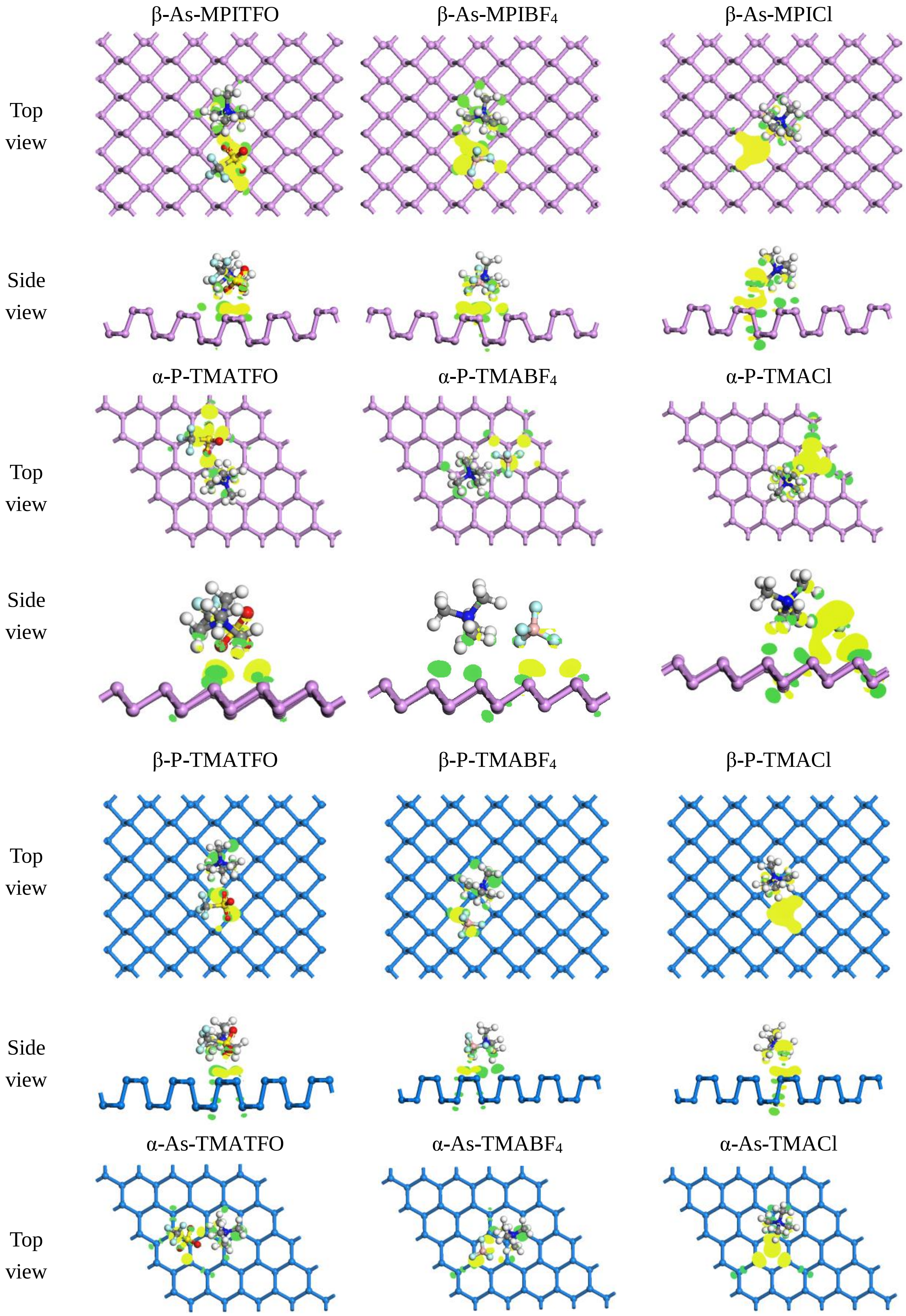
β-As-MPITFO
β-As-MPIBF$_4$
β-As-MPICl
Top view
Side view
α-P-TMATFO
α-P-TMABF$_4$
α-P-TMACl
Top view
Side view
β-P-TMATFO
β-P-TMABF$_4$
β-P-TMACl
Top view
Side view
α-As-TMATFO
α-As-TMABF$_4$
α-As-TMACl
Top view

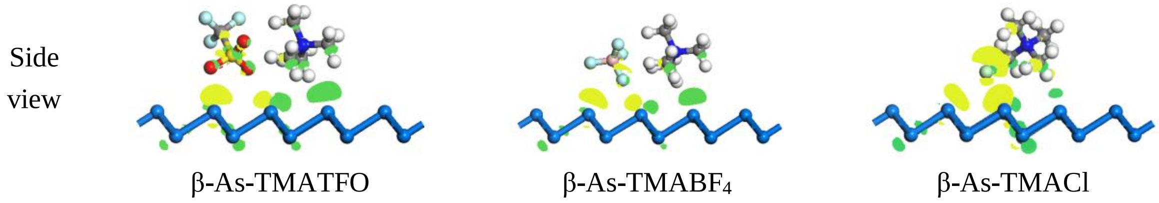

Figure S5. Top and side views of differential electron density of ILs([MPI][TFO], [MPI][BF4], [MPI][Cl], [TMA][TFO], [TMA][BF$_4$], and[TMA][Cl]) adsorbed on α-P, β-P, α-As, and β-As nanosheets. Green and yellow areas correspond to accumulation and depletion of electronic densities, respectively, with an isosurface value of 0.003e/Å^3.

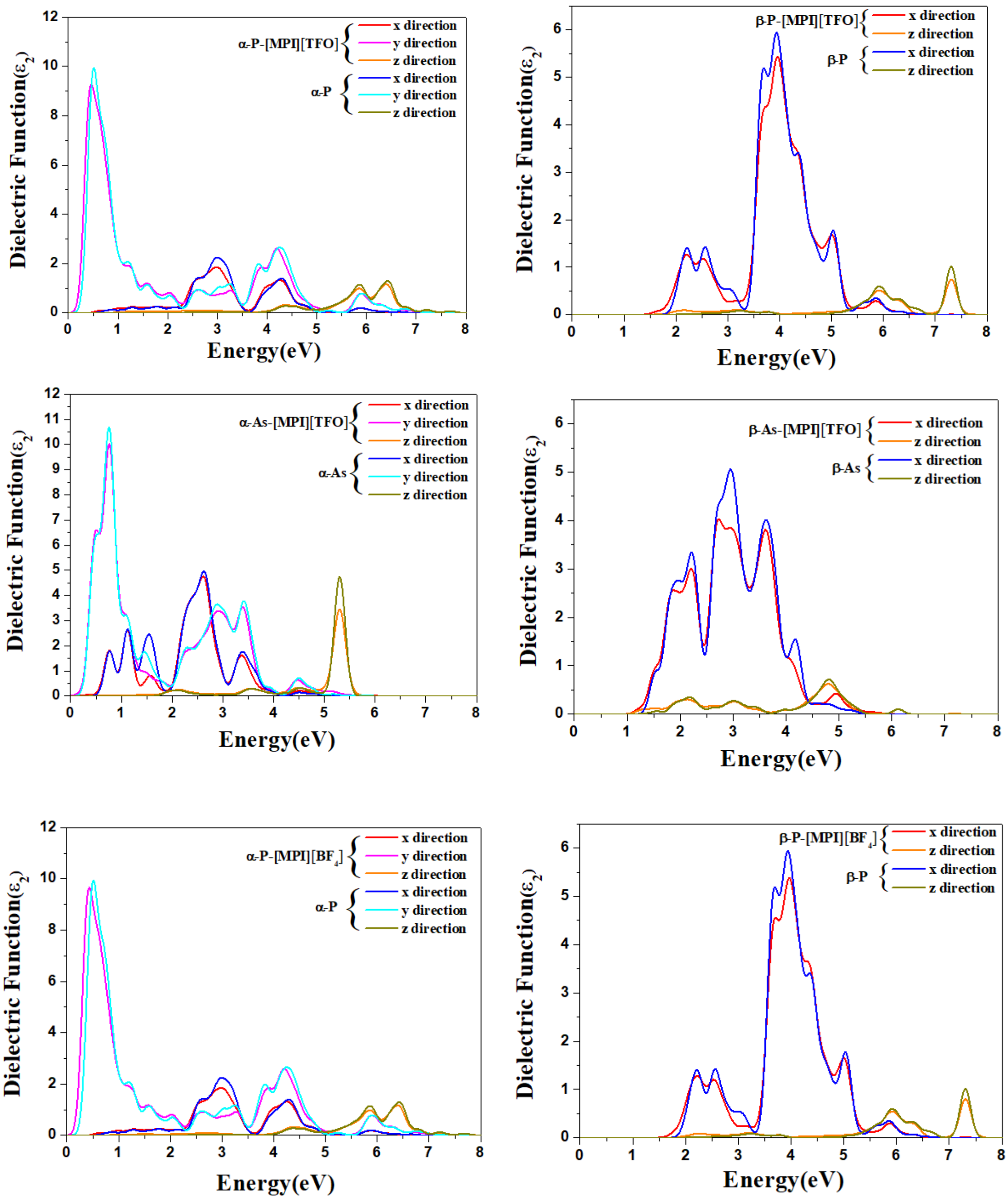

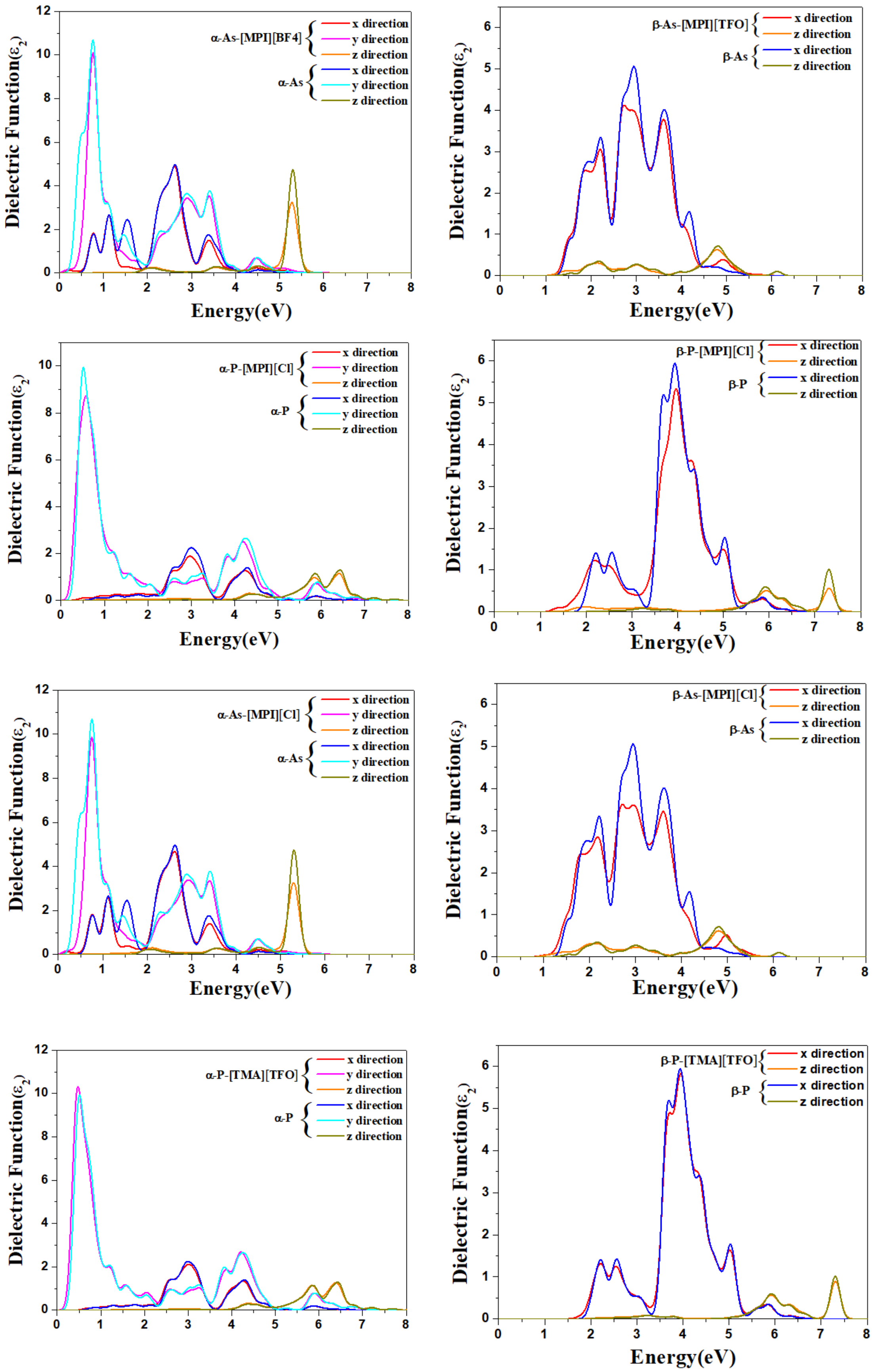

α-As-[MPI][BF4]
α-As
x direction
y direction
z direction
Dielectric Function(ε2)
Energy(eV)
β-As-[MPI][TFO]
β-As
x direction
z direction
Dielectric Function(ε2)
Energy(eV)
α-P-[MPI][Cl]
α-P
x direction
y direction
z direction
Dielectric Function(ε2)
Energy(eV)
β-P-[MPI][Cl]
β-P
x direction
z direction
Dielectric Function(ε2)
Energy(eV)
α-As-[MPI][Cl]
α-As
x direction
y direction
z direction
Dielectric Function(ε2)
Energy(eV)
β-As-[MPI][Cl]
β-As
x direction
z direction
Dielectric Function(ε2)
Energy(eV)
α-P-[TMA][TFO]
α-P
x direction
y direction
z direction
Dielectric Function(ε2)
Energy(eV)
β-P-[TMA][TFO]
β-P
x direction
z direction
Dielectric Function(ε2)
Energy(eV)

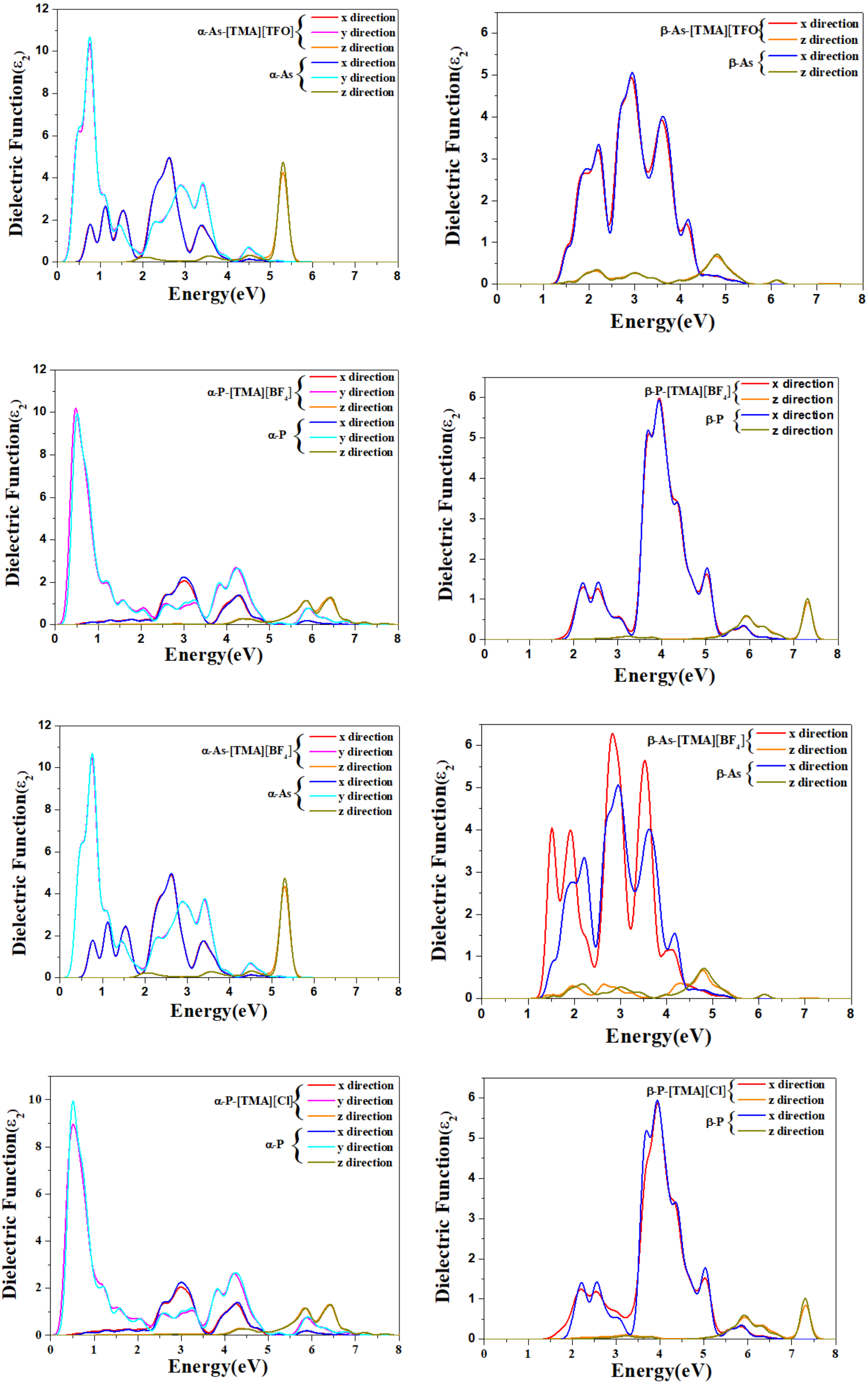

α-As-[TMA][TFO]
x direction
y direction
z direction
α-As
x direction
y direction
z direction
Dielectric Function(ε_2)
Energy(eV)
β-As-[TMA][TFO]
x direction
z direction
β-As
x direction
z direction
Dielectric Function(ε_2)
Energy(eV)
α-P-[TMA][BF_4]
x direction
y direction
z direction
α-P
x direction
y direction
z direction
Dielectric Function(ε_2)
Energy(eV)
β-P-[TMA][BF_4]
x direction
z direction
β-P
x direction
z direction
Dielectric Function(ε_2)
Energy(eV)
α-As-[TMA][BF_4]
x direction
y direction
z direction
α-As
x direction
y direction
z direction
Dielectric Function(ε_2)
Energy(eV)
β-As-[TMA][BF_4]
x direction
z direction
β-As
x direction
z direction
Dielectric Function(ε_2)
Energy(eV)
α-P-[TMA][Cl]
x direction
y direction
z direction
α-P
x direction
y direction
z direction
Dielectric Function(ε_2)
Energy(eV)
β-P-[TMA][Cl]
x direction
z direction
β-P
x direction
z direction
Dielectric Function(ε_2)
Energy(eV)

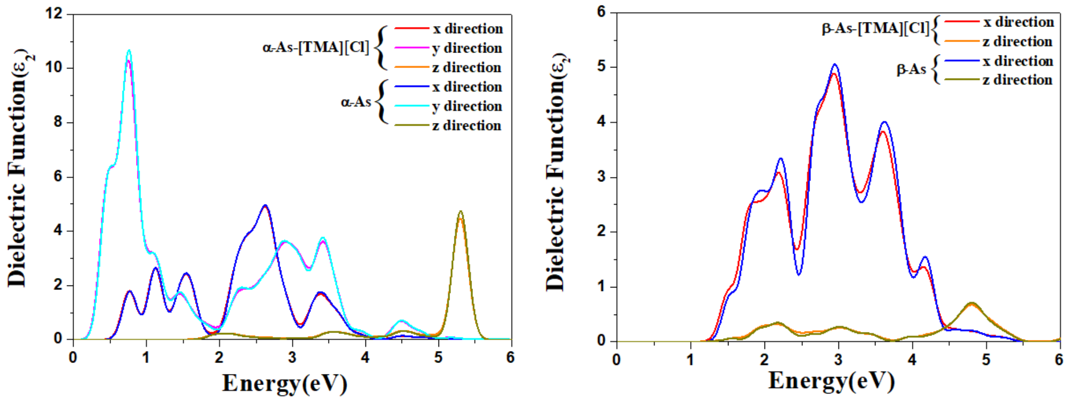

Figure S6. Computed imaginary dielectric functions versus energy for isolated and complexes of ILs([MPI][TFO], [MPI][BF_4], [MPI][Cl], [TMA][TFO], [TMA][BF_4], and [TMA][Cl]) adsorbed on α-P and α-As in the x, y, z direction, and β-P and β-As in the x, z direction.

Table S2. The Mulliken and Hirshfeld charge difference of ionic liquids (ILs) and nanosheets after adsorption (values in e).

Structures	Mulliken charge			Hirshfeld Charge		
	$\Delta q(+)^a$	$\Delta q(-)^b$	Δq_{sur}	$\Delta q(+)^a$	$\Delta q(-)^b$	Δq_{sur}
α-P-EMIMTFO	0.003	0.094	-0.095	-0.248	0.102	0.147
β-P-EMIMTFO	-0.016	0.096	-0.080	-0.180	0.064	0.116
α-As-EMIMTFO	-0.040	0.045	-0.007	-0.177	0.075	0.104
β-As-EMIMTFO	-0.084	0.060	0.018	-0.208	0.087	0.121
α-P-EMIMBF_4	0.001	0.073	-0.072	-0.186	0.065	0.124
β-P-EMIMBF_4	-0.018	0.085	-0.067	0.261	0.073	0.188
α-As-EMIMBF_4	-0.057	0.067	-0.012	-0.207	0.087	0.122
β-As-EMIMBF_4	-0.078	0.043	0.032	-0.219	0.050	0.167
α-P-EMIMCl	0.252	0.108	-0.359	0.029	0.143	-0.163
β-P-EMIMCl	0.234	0.159	-0.389	0.009	0.172	-0.182
α-As-EMIMCl	0.170	0.047	-0.214	-0.013	0.092	-0.079
β-As-EMIMCl	0.160	0.090	-0.250	0.028	0.125	-0.155
α-P-MPITFO	-0.016	0.072	-0.058	-0.286	0.124	0.162
β-P-MPITFO	-0.014	0.110	-0.096	-0.221	0.139	0.084
α-As-MPITFO	-0.168	0.031	-0.139	-0.327	0.115	0.214
β-As-MPITFO	-0.103	0.037	0.065	-0.258	0.118	0.139
α-P-MPIBF_4	-0.042	0.082	-0.041	-0.255	0.077	0.177
β-P-MPIBF_4	-0.021	0.077	-0.057	-0.205	0.066	0.139
α-As-MPIBF_4	-0.183	0.043	0.141	-0.286	0.076	0.212
β-As-MPIBF_4	-0.134	0.061	0.074	-0.270	0.101	0.168
α-P-MPICl	0.358	-0.04	-0.323	0.106	0.032	-0.137
β-P-MPICl	0.392	-0.012	-0.384	0.189	0.047	-0.236
α-As-MPICl	0.161	-0.101	-0.064	0.022	-0.027	0.006

β-As-MPICl	0.270	-0.055	-0.214	0.131	0.005	-0.137
α-P-TMATFO	0.027	0.061	-0.083	-0.124	0.047	0.079
β-P-TMATFO	0.004	0.080	-0.083	-0.118	0.043	0.075
α-As-TMATFO	-0.016	0.045	-0.029	-0.133	0.082	0.053
β-As-TMATFO	-0.051	0.054	-0.003	-0.089	0.055	0.088
α-P-$TMABF_4$	0.006	0.065	-0.072	-0.172	0.030	0.144
β-P-$TMABF_4$	-0.006	0.070	-0.062	-0.129	0.016	0.113
α-As-$TMABF_4$	-0.038	0.045	-0.005	-0.161	0.051	0.113
β-As-$TMABF_4$	-0.053	0.038	0.015	-0.155	0.046	0.108
α-P-TMACl	0.149	0.202	-0.354	0.004	0.201	-0.202
β-P-TMACl	0.148	0.231	-0.380	0.001	0.216	-0.218
α-As-TMACl	0.098	0.129	-0.227	-0.030	0.152	-0.121
β-As-TMACl	0.088	0.146	-0.234	-0.015	0.171	-0.157

*$\triangle q\ (+)^a = q_{\text{cation in IL (after adsorption)}} - q_{\text{cation in IL (before adsorption)}}$;

*$\triangle q\ (-)^b = q_{\text{anion in IL (after adsorption)}} - q_{\text{anion in IL (before adsorption)}}$.
